# Biomimetic Metal–Organic Framework Gated Nanoplatform for Sonodynamic Therapy against Extensively Drug Resistant Bacterial Lung Infection

**DOI:** 10.1002/advs.202402473

**Published:** 2024-07-04

**Authors:** Jianling Huang, Xiuwen Hong, Sixi Chen, Yucong He, Lixu Xie, Fenglin Gao, Chenghua Zhu, Xiao Jin, Haihao Yan, Yongxia Ye, Mingyue Shao, Xingran Du, Ganzhu Feng

**Affiliations:** ^1^ Department of Pulmonary and Critical Care Medicine The Second Affiliated Hospital of Nanjing Medical University Nanjing Jiangsu 210011 China; ^2^ Department of Pulmonary and Critical Care Medicine Qi Lu Hospital of Shandong University Wen hua xi Road 107# Jinan 250012 China; ^3^ Department of Radiology Nanjing Medical University Affiliated Cancer Hospital Jiangsu Cancer Hospital Jiangsu Institute of Cancer Research Nanjing Jiangsu 210009 China; ^4^ Department of Respiratory and Critical Care Medicine The Affiliated Jiangning Hospital with Nanjing Medical University Nanjing 211100 China

**Keywords:** biomimetic nanoplatform, extensively drug resistant bacteria, mesenchymal stem cells, metal–organic framework, polymyxin B, sonodynamic therapy

## Abstract

Novel antimicrobial strategies are urgently needed to treat extensively drug‐resistant (XDR) bacterial infections due to the high mortality rate and lack of effective therapeutic agents. Herein, nanoengineered human umbilical cord mesenchymal stem cells (hUC‐MSCs), named PMZMU, are designed as a sonosensitizer for synergistic sonodynamic‐nano‐antimicrobial therapy against gram‐negative XDR bacteria. PMZMU is composed of a bacterial targeting peptide (UBI_29‐41_) modified hUC‐MSCs membrane (MSCm), a sonosensitizer meso‐tetra(4‐car‐boxyphenyl) porphine doped mesoporous organo‐silica nanoparticle and an acidity‐responsive metal–organic framework ZIF‐8. This innovative formulation enables efficient loading of polymyxin B, reduces off‐target drug release, increases circulation and targeting efficacy, and generates reactive oxygen species upon ultrasound irradiation. PMZMU exhibits remarkable in vitro inhibitory activity against four XDR bacteria: *Klebsiella pneumoniae, Acinetobacter baumannii, Pseudomonas aeruginosa* (PA), and *Escherichia coli*. Taking advantage of the bacterial targeting ability of UBI_29‐41_ and the inflammatory chemotaxis of hUC‐MSC, PMZMU can be precisely delivered to lung infection sites thereby augmenting polymyxin B concentration. PMZMU‐mediated sonodynamic therapy significantly reduces bacterial burden, relieves inflammatory damage by promoting the polarization of macrophages toward M_2_ phenotype, and improves survival rates without introducing adverse events. Overall, this study offers promising strategies for treating deep‐tissue XDR bacterial infections, and guides the design and optimization of biomimetic nanomedicine.

## Graphical abstract

PMZMU is designed as a sonosensitizer for synergistic sonodynamic‐nano‐antimicrobial therapy against gram‐negative XDR bacteria. PMZMU is composed of a bacterial targeting peptide (UBI_29‐41_) modified hUC‐MSCs membrane (MSCm), a sonosensitizer doped mesoporous organo‐silica nanoparticle, and an acidity‐responsive metal–organic framework. PMZMU‐mediated SDT significantly reduces bacterial burden, relieves inflammatory damage, and improves survival rates without introducing adverse events.

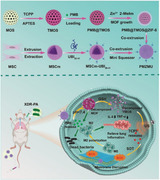

## 1 Introduction

Resistant to nearly all available antibiotics, the rapid evolution and spread of extensively drug‐resistant (XDR) gram‐negative bacteria have significantly impacted conventional antimicrobial stewardship, posing a significant challenge to clinical treatment.^[^ ^1^ ^]^ The development of novel antimicrobial drugs, although imminent, is constrained by numerous obstacles, including protracted development times, exorbitant costs, and stringent regulatory hurdles.^[^ ^2^ ^]^ One strategy to address this situation is to revitalize traditional antibiotics through modification and enhancement. Polymyxin B (PMB) serves as the ultimate defense against XDR bacteria, effectively disrupting bacterial cell membrane permeability by binding its polycationic ring to phosphate groups, resulting in bacterial swelling and lysis. However, the insufficient concentration of PMB in lung tissues and potential side effects such as allergic reactions, nephrotoxicity, and neurotoxicity limit its widespread use in clinical practice.^[^ ^3^, ^4^, ^5^ ^]^ Hence, strategies are needed to elevate the concentration of PMB in lung tissue and reduce its toxicity for the treatment of XDR gram‐negative bacterial lung infections. The remarkable advancements of nanotechnology may be the key to improve the therapeutic properties of existing antibiotics.^[^ ^6^ ^]^ Nanoparticle carriers can target drug delivery to the infection site, and extend the circulation time while protecting the drug from degradation or inactivation in body fluids and the gastrointestinal tract. Furthermore, immobilization of drugs in nanoparticles can reduce adverse side effects.^[^ ^7^, ^8^ ^]^ Among these, redox‐responsive biodegradable disulfide‐bond‐bridged mesoporous organo‐silica (MOS) nanoparticles are particularly noteworthy due to their large surface area, interpenetrating pore channels, excellent biocompatibility, and their utilization of glutathione as the stimulus for drug release.^[^ ^9^, ^10^, ^11^ ^]^ As a biologically effective cleavable linker, the disulfide bonds in the framework of carrier materials have achieved their fragmentation and degradation in the reducing medium.^[^ ^12^ ^]^ Compared to traditional mesoporous silica nanoparticles, MOS exhibited faster drug release rate and higher potential for clinical translation.^[^ ^10^ ^]^ The mesoporous structure of MOS could be successfully used for efficient drug loading and delivery. However, the spontaneous or off‐target release of small drug molecules remains a significant challenge for MOS‐based nanocarriers, necessitating the development of gatekeepers to seal the pore channels of MOS to prevent premature drug leakage.^[^ ^13^ ^]^

Metal–organic frameworks (MOFs) are 3D porous crystalline materials self‐assembled from metal ions or metal clusters with organic ligands via coordination bonds, which have the potential to act as gatekeepers to prevent drug leakage. MOFs can overcome the limitations of pore entry and gatekeeper size matching and decorate the surface of MOS nanoparticles uniformly, yielding excellent pore‐plugging effects.^[^ ^13^ ^]^ Zeolite imidazole framework‐8 (ZIF‐8) is an acid‐responsive MOF that remains stable in neutral physiological environments but degrades rapidly under acidic conditions, allowing selective drug release in infected microenvironments (IMEs) characterized by low pH, bacterial enzymes, and activated blood vessels.^[^ ^14^, ^15^, ^16^, ^17^ ^]^ Therefore, the outer coating of ZIF‐8 on MOS effectively seals its pores, preventing drug leakage into normal tissues, while enabling pH‐responsive, controlled drug release in infected microenvironments. Nevertheless, exogenous MOS‐based nanomaterials lack an active inflammation‐targeting mechanism and are easily recognized and cleared by macrophages upon entering the organism, thereby affecting their targeted delivery efficacy and potentially triggering an immune response.^[^ ^9^ ^]^ It is particularly important to develop antimicrobial drug nano‐delivery systems that are inflammation‐targeted and avoid clearance during blood circulation.

Cell membrane‐coated nanoparticles (CM‐NPs), often referred to as biomimetic nanoparticles, have attracted widespread attention in drug delivery and garnered significant attention in recent years.^[^ ^18^ ^]^ By combining some of the best features of both naturally occurring membranes and synthetic core materials, CM‐NPs possess unique features, such as immunological invasion, prolonged circulation in vivo, and active targeting capacity.^[^ ^19^ ^]^ Currently, various cell membranes, such as those of erythrocytes, macrophages, and neutrophils, have been successfully employed for the preparation of CM‐NPs.^[^ ^20^ ^]^ The distinct compositions of proteins, glycans, and lipids in cell membranes from various sources lead to diverse functionalities. Mesenchymal stem cells (MSCs) are stem cells with self‐renewal and multidirectional differentiation potentials, characterized by inflammatory chemotaxis, immunomodulation, and low immunogenicity.^[^ ^21^ ^]^ Encapsulation of MSC membranes onto nanoparticles has been reported to increase biocompatibility, decrease immunogenicity, prolong circulation, and promote pathogen targeting.^[^ ^22^, ^23^ ^]^ Human umbilical cord mesenchymal stem cells (hUC‐MSCs) not only retain the biological properties of MSCs but also offer additional advantages such as ease of access, robust proliferative capacity, and lower immunogenicity.^[^ ^24^ ^]^ These features provide a plentiful source of cell membranes for the construction of biomimetic nanoparticles.

Compared to monotherapy, synergistic therapies are more advantageous in terms of therapeutic efficiency and safety. In recent years, a variety of antibiotics‐alternative antibacterial strategies have been developed based on nanotechnology, such as phototherapy,^[^ ^25^ ^]^ sonodynamic therapy (SDT),^[^ ^26^ ^]^ and chemo‐dynamic therapy,^[^ ^27^ ^]^ characterized by their ability to destroy bacteria while being less likely to develop antibiotic resistance. Among them, SDT is a noninvasive antibacterial approach that utilizes low‐intensity ultrasound (US) to activate sonosensitizers, thereby generating reactive oxygen species (ROS) to eliminate bacteria.^[^ ^28^ ^]^ By leveraging the remarkable tissue permeability and targeted irradiation of ultrasound, SDT offers a hopeful solution for deep‐seated infections without bringing systemic toxicity.^[^ ^29^ ^]^ To date, SDT has received significant research interest, and numerous sonosensitizers including meso‐tetra(4‐carboxyphenyl) porphine (TCPP), single‐atom Pt catalyst, TiO_2_, and red phosphorus have been effectively utilized in the elimination of drug resistant bacteria.^[^ ^30^, ^31^, ^32^, ^33^ ^]^ However, the inherent drawbacks of most sonosensitizers, including poor aqueous solubility, low bioavailability, and lack of pathogens specificity, compromise the effectiveness of SDT.^[^ ^34^ ^]^ The combination of nano‐delivery systems with alternative therapies may be an effective way to overcome these drawbacks. It has been reported MSC membrane‐encapsulated nanoparticles can be utilized in conjunction with SDT to partially overcome the limitations of sonosensitizers, thereby improving the therapeutic effectiveness of SDT.^[^ ^35^ ^]^ However, such studies have focused on tumor therapy, and no studies are reporting its application in drug‐resistant bacterial infections.

**Scheme 1:**
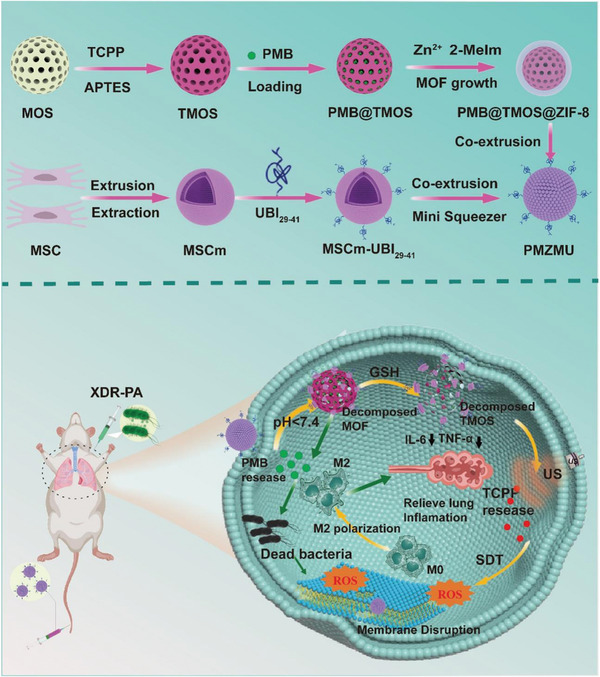
Preparation of PMZMU nanoparticles and schematic illustration of sonodynamic therapy for improving antimicrobial efficacy of XDR bacterial pneumonia. APTES, (3‐Aminopropyl) triethoxysilane; Zn^2+^, Zn (NO_3_)_2_·6H_2_O; 2‐Melm,2‐methylimidazole.

Here, we developed a novel biomimetic nanoplatform PMB@TMOS@ZIF‐8@MSCm‐UBI_29‐41_ (PMZMU), specifically designed for targeted delivery of antimicrobial drugs and sonodynamic therapy. This platform comprised of a TCPP‐doped mesoporous organo‐silica (TMOS) nanoparticle loaded with PMB, sealed with a metal–organic framework, ZIF‐8, as acidity‐responsive gatekeeper, and further camouflaged with UBI_29‐41_ peptides modified MSC membrane (MSCm) exhibiting inflammatory targeting and immune‐evading properties. In neutral tissues, ZIF‐8 remained stable and effectively prevented leakage of PMB from the TMOS. Upon reaching the bacterial microenvironment, the MOF degraded rapidly, enabling the controlled release of PMB. Activated by ultrasound, the remaining TMOS triggered TCPP‐mediated SDT for eliminating bacteria and biofilms, and achieved self‐degradation by its disulfide bond (─S─S─) reacting with glutathione (GSH) within the BME. The in vitro and in vivo studies validated the enhanced bactericidal activity and favorable biosafety of the nanoplatform. In an XDR‐PA pneumonia mouse model, we demonstrated that intravenous administration of PMZMU specifically targeted the lung, effectively eliminated XDR‐PA load, relieved pro‐inflammatory response by regulating M_2_ macrophage polarization, and improved survival rate under deep‐penetrating ultrasound irradiation (**Scheme** **1**). Therefore, our study illuminates the integration of biomimetic nano‐delivery systems with SDT to overcome major limitations and drawbacks in the treatment of XDR bacterial lung infections, and provides insights into the design and optimization of biomimetic nano‐antimicrobial drugs.

## 2 Results and Discussion

### 2.1 Synthesis and Characterization of TMOS and TMOS@ZIF‐8

**Figure 1:**
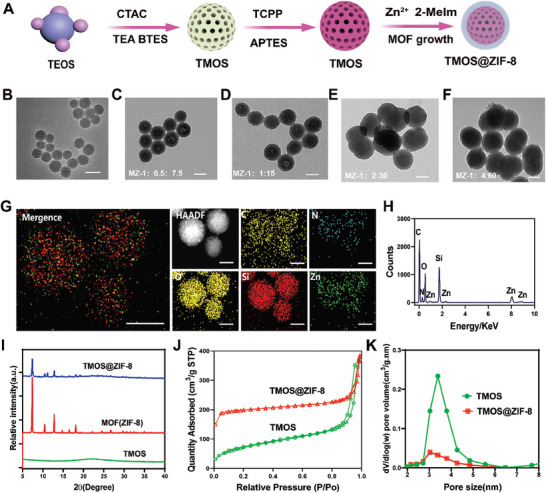
Structure of TMOS and TMOS@ZIF‐8. A) Scheme for the synthesis of TMOS and TMOS@ZIF‐8. B) Representative TEM image of TMOS. Scale bar: 50 nm. C–F) Representative TEM images of TMOS@ZIF‐8 with different TMOS to Zn (NO_3_)_2_ to 2‐MeIm. Scale bar: 50 nm. G) Energy‐dispersive X‐ray spectroscopy (EDS) spectrum element mapping of TMOS@ZIF‐8. H) EDS spectrum of TMOS@ZIF‐8. All the major elements (C, N, O, Si, and Zn) can be found in the spectrum. I) The X‐ray diffraction patterns of TMOS, ZIF‐8, and TMOS@ZIF‐8. J) N_2_ adsorption‐desorption isotherms of TMOS and TMOS@ZIF‐8. (K) Pore size distributions of TMOS and TMOS@ZIF‐8 nanoparticles.

Small‐sized mesoporous organo‐silica (MOS) nanoparticles were synthesized via the hydrolysis of tetraethyl orthosilicate (TEOS) under the structural direction of cetyltrimethylammonium chloride (CTAC), followed by the deposition of a MOS shell on mesoporous silica to yield MOS through the co‐hydrolysis of bis[3‐(triethoxysilyl) propyl] tetrasulfide (BTES) and TEOS (**Figure** **1**A). After doped TCPP into the backbone of MOS, MOS solution turned red (Figure S1A, Supporting Information). UV–vis absorbance spectrometry also showed the successful doping of TCPP, and the doping percentage of TCPP in TMOS was 12.2% (Figure S1B,C, Supporting Information). Transmission electron microscopy (TEM) images show that the TMOS nanoparticles are highly dispersed and uniformly spherical, with an average diameter of ≈50 nm (Figure 1B). TMOS@ZIF‐8 nanocomposites with different TMOS to MOF mass ratios were synthesized by mixing TMOS with solutions containing Zn^2+^ for 15 min of sonication at ice bath, followed by addition of 2‐MeIm for another 15 min of sonication. The negative potential of TMOS (Figure S2, Supporting Information) enabled positive charged Zn^2+^ to easily precipitate on the surface and within the channels of TMOS. Additionally, the heterogeneous structure of TMOS@ZIF‐8 could be tuned by adjusting the mass ratio of TMOS to MOF precursors [Zn (NO_3_)_2_ and 2‐MeIm]. When a high concentration of TMOS reacted with low concentrations of Zn (NO_3_)_2_ and 2‐MeIm (1:0.5:7.5), the mesopores of TMOS could still be observed in the TEM image (Figure 1C), indicating that the formed MOF component was not sufficient to seal the pore channels. As the concentrations of Zn (NO_3_)_2_ and 2‐MeIm increased (1:1:15), there was an enhanced MOF formation that led to effectively sealing the pores of TMOS (Figure 1D). With the increase of the mass of the MOF precursor, the MOF layer on the TMOS surface was subsequently thickened, which ultimately led to the aggregation of nanomaterials (Figure 1E). When the amount of MOF component was much higher than the mass of TMOS, the MOF component would not be encapsulated on the surface of the TMOS, and instead, homogeneous nucleation occurs, and the excess MOF component forms individual nanoparticles (Figure 1F). After carefully considering all the relevant factors mentioned above, we selected TMOS@ZIF‐8 1:1:15 as the nanocarrier for further use. Dynamic light scattering (DLS) measurements showed that the hydrodynamic size of the resulting TMOS@ZIF‐8 nanoparticles increased to 101.32 ± 3.47 nm (Figure S3A, Supporting Information) from 68.28 ± 3.89 nm (Figure S3B, Supporting Information). Element mapping and energy‐dispersive X‐ray spectroscopy (EDS) analysis confirmed the presence of C, N, O, Si, S, and Zn elements, with a uniform distribution of the Zn component on the outer surface of TMOS@ZIF‐8 (Figure 1G,H). X‐ray photoelectron spectroscopy (XPS) spectrum revealed two distinct binding energies of Zn 2p at 1041.38 and 1022.38 eV in TMOS@ZIF‐8 (Figure S4, Supporting Information). The X‐ray diffraction pattern of TMOS@ZIF‐8 also exhibited similar characteristic peaks as ZIF‐8(Figure 1I). All these findings confirmed successful ZIF‐8 modification on TMOS surface. According to Brunauer‐Emmett‐Teller (BET) results, TMOS@ZIF‐8 displayed a large specific surface area of 616.47 m^2^ g^−1^, facilitating drug loading and delivery (Figure 1J). The pore volume of TMOS@ZIF‐8 was significantly decreased compared to TMOS (8.82 nm vs 3.86 nm), suggesting successful MOF sealing in the pore channels of TMOS (Figure 1K).

### 2.2 Synthesis and Characterization of TMOS@ZIF‐8@MSCm (MZM) and TMOS@ZIF‐8@MSCm‐UBI (MZMU)

**Figure 2:**
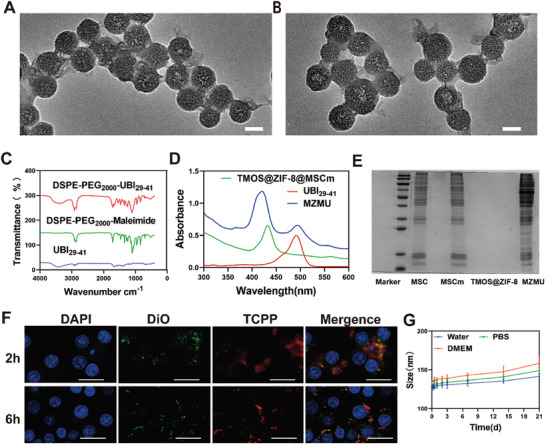
Fabrication and characterization of MZMU. A) Representative TEM image of MZM. Scale bar:50 nm. B) Representative TEM images of MZMU. Scale bar: 50 nm. C) FTIR spectra of DSPE‐PEG_2000_‐Maleimide, UBI_29‐41_ and DSPE‐PEG_2000_‐ UBI_29‐41_. D) UV‐vis spectra of UBI_29‐41_ peptide, MZM and MZMU. E) Images of SDS‐PAGE gels to examine protein contents of whole mesenchymal stem cells (MSC), mesenchymal stem cell membranes (MSCm), TMOS@ZIF‐8 nanoparticles and MZMU nanoparticles. F) Confocal fluorescence images corresponding to co‐localization of DiO‐labeled MSC membrane modifications (DiO, green) and TMOS@ZIF‐8 nanoparticles (TCPP, red) in RAW264.7 cells incubated with 25 µg mL^−1^ MZMU nanoparticles for 2 h and 6 h. Scale bar:20 µm. G) Particle size of MZMU at different time points determined by DLS measurements. MZMU was dispersed in water, pH 7.4 PBS and DMEM at 4 °C for 21 days. (n = 3, Data are expressed in mean ± SD).

To achieve inflammation targeting and evasion from the immune system for in vivo drug delivery, MSC membrane‐cloaked NPs were fabricated by coextruding MSC membrane fragments and TMOS@ZIF‐8 nanoparticles. UBI_29‐41_ and fluoresceine isothiocyanate (FITC)‐labeled UBI_29‐41_ peptides were synthesized and then conjugated with DSPE‐PEG_2000_‐Maleimide to obtain DSPE‐PEG_2000_‐UBI_29‐41_, which could be spontaneously incorporated onto cell membranes via a lipid‐insertion approach. The coating with MSCm formed thin and smooth surrounding layer on the TMOS@ZIF‐8 (**Figure** **2**A), increased the size of the nanoparticles to ≈118 nm (Figure S5, Supporting Information) and decreased the zeta potential from ≈−30 to ≈−40 mV (Figure S2, Supporting Information). UBI_29‐41_ peptide modification to the outer surface of MZM resulted in a more dispersed and homogeneous distribution of nanoparticles (Figure 2B). The average hydrodynamic size of MZMU nanoparticles increased to 129.88 ± 4.55 nm (Figure S6, Supporting Information), probably due to the additional PEG chains incorporated onto MSC membranes after MZM modified by DSPE‐PEG_2000_‐UBI_29‐41_. Fourier transform infrared spectroscopy (FTIR) results showed a characteristic absorption peak at 1986 cm^−¹^ for the sulfide‐ether bond (S─O) (Figure 2C). After conjugating the FITC‐labeled UBI_29‐41_ peptide to the outer surface of MZM, the FITC peak was observed at 490 nm using UV–vis absorbance spectroscopy (Figure 2D). Sodium dodecyl sulfate polyacrylamide gel electrophoresis (SDS‐PAGE) results indicated that the membrane proteins from the source cell membrane could be well retained on the MZMU after coating (Figure 2E). MSC membrane, labeled with a green fluorescent dye (DiO), was coated onto preformed TMOS@ZIF‐8 cores, immobilized in glycerol, and observed under confocal microscopy, revealing significant colocalization of fluorescent signals (Figure S7, Supporting Information). The high degree of intracellular co‐localization between the TMOS@ZIF‐8 and cell membranes after 2 h of uptake further verified the structural integrity of the MZMU (Figure 2F). All these results confirmed the successful construction of MZMU nanoparticles. In addition, MZMU exhibited good colloidal stability and dispersibility after 21 days incubation in distilled water, pH7.4 phosphate buffer solution (PBS), and DMEM medium (Figure 2G). To verify their immune invasion capacity, an evaluation of macrophage uptake of MZMU was conducted. Notably, RAW264.7 cells internalized a significant amount of TMOS@ZIF‐8, exhibiting intense fluorescence. However, coating with the MSC membrane significantly reduced fluorescence intensity, indicating its potential to inhibit immune cell recognition and invasion (Figure S8, Supporting Information).

### 2.3 Sonodynamic Activity of MZMU

Single‐linear oxygen (^1^O2) is recognized as a primary active species of ROS and a key mediator in the sensitizer cytotoxicity mechanism based on the porphyrin structure.^[^ ^36^ ^]^ To evaluate the capacity of MZMU to generate ^1^O2 under US irradiation, Electron Spin Resonance (ESR) experiments were conducted, utilizing 2,2,6,6‐tetramethylpiperidine (TEMP) as a spin‐trapping agent. The three peaks with a 1:1:1 ratio, as displayed in Figure S9 (Supporting Information), represent the signatures of ^1^O2. Subsequently, 9,10‐Anthracenediylbis(methylene) dipropanedioic acid (ABDA) was employed as a probe to assess the capacity of MZMU to generate ^1^O2 under US irradiation. This choice was based on the fact that ABDA can be oxidized by ^1^O2 to produce the corresponding endoperoxide, resulting in a reduction of the UV absorption peak at 400 nm (Figure S10, Supporting Information). Subsequently, 9,10‐, the peak intensity of ABDA at 400 nm decreased as the US irradiation time increased from 0 to 30 min, indicating that the production of ^1^O2 by MZMU under US stimulation was time‐dependent.

### 2.4 pH‐Dependent Nanoparticle Decomposition and Drug Release Behavior

**Figure 3:**
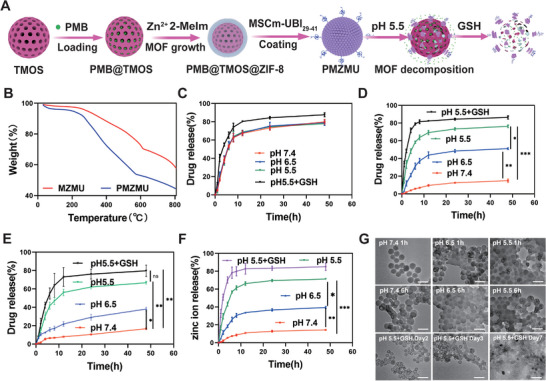
Dual‐responsive degradation and drug release behavior of PMZMU. A) Schematic for the PMB release profiles of PMZMU at different pH values and GSH‐containing medium. B) Thermogravimetric analysis (TGA) curves of MZMU and PMZMU. PMB release profiles of C) PMB@TMOS, D) PMZ, and E) PMZMU at different pH values and GSH‐containing medium. (n = 3, ^*^ *p* <0.05, ^**^ *p* <0.01, ^***^ *p* <0.001. Data are expressed in mean ± SD). F) Zinc ion release profiles of PMZMU at different pH values and GSH‐containing medium. (n = 3, ^*^ *p* <0.05, ^**^ *p* <0.01, ^***^ *p* <0.001. Data are expressed in mean ± SD). G) Representative TEM images showing the pH and GSH dual responsive degradation of PMZMU nanoparticles. Scale bar:100 nm.

TMOS was mixed with PMB at a 1:2 ratio under stirring overnight to fabricate PMB‐loaded nanoshell. Subsequently, the mixture was sequentially coated with MOF and cell membrane (**Figure** **3**A). The results of thermogravimetric analysis (TGA) indicated weight loss values of 42.32% and 55.67% for MZMU and PMB@TMOS@ZIF‐8@MSCm‐UBI_29‐41_ (PMZMU) nanoparticles, respectively, indicating a PMB content of 13.35% (Figure 3B). Without the MOF gate, the loaded drugs easily leaked from the TMOS, displaying similar PMB release profiles at different pH values. Nearly 80% of the loaded PMB was continuously released from PMB@TMOS within 24 h (Figure 3C). In contrast, PMB@TMOS@ZIF‐8 (PMZ) exhibited distinct drug release behaviors under various pH conditions. In neutral solution, it showed a slow release of PMB, which was attributed to the stable MOF coating blocking the pores of TMOS and significantly reducing drug leakage. Due to the acidity‐activated degradation of MOF, the pores of TMOS were uncovered, inducing about nearly 50% and 70% PMB release at pH 6.5 and 5.5 within 12 h, respectively (Figure 3D). Moreover, PMB release from PMZMU was slower than that from PMZ, possibly due to the MSCm barriers (Figure 3E). In the GSH‐containing medium, nanoparticles showed a higher drug release trend which may be attributed to TMOS degradation in the presence of GSH. Similar to PMB, Zn^2+^ in PMZMU also exhibited pH‐responsive release behavior. Figure 3F showed a robust release of nearly 70% of Zn^2+^ at pH 5.5 within 12 h, whereas a much slower release of less than 15% Zn^2+^ was observed at pH 7.4 over 48 h. Notably, we also found that GSH accelerated the release of zinc ions. This suggests that GSH not only triggers the degradation of TMOS but also promotes the catabolism of ZIF‐8, which is consistent with the study reported by Du et al.^[^ ^37^ ^]^ Moreover, the morphological changes of PMZMU under different pH values were also observed using TEM. After incubating PMZMU in pH 5.5 PBS for 1 h, the outer MOF structure began to decompose and was completely decomposed after 6 h. When incubated in pH 5.5 PBS containing GSH (5 mm) for 48 h, TMOS started to degrade, including surface and bulk erosion and eventually disintegrated into fragments over one week (Figure 3G). These phenomena are likely caused by the disulfide bond cleavage under both reducing conditions.

### 2.5 In Vitro Antibacterial Activity and Mechanisms of PMZMU Against Pathogens Under US Irradiation

**Figure 4:**
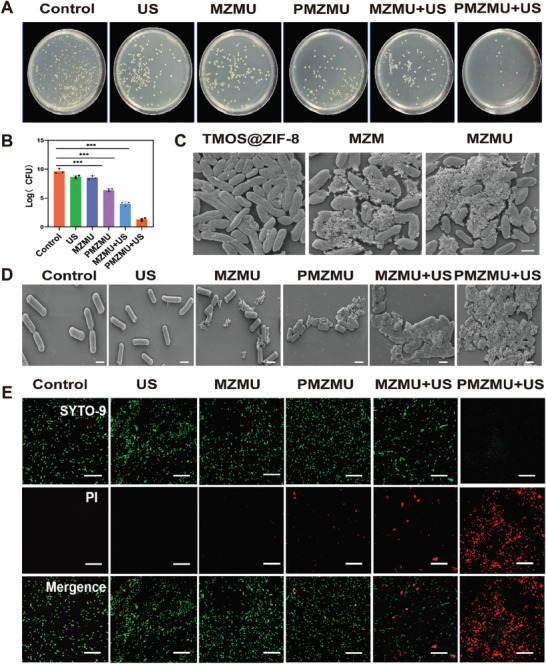
The antibacterial activity of PMZMU mediated SDT in vitro. A) Typical Photographs and B) strain counts of extensively drug‐resistant *P. aeruginosa* (XDR‐PA) calculated from spread‐plate assays after treated with PBS (control), US, MZMU, PMZMU, MZMU+US and PMZMU+US (n = 3, ^***^ *p* <0.001. Data are expressed in mean ± SD). C) SEM image of XDR‐PA incubated with TMOS@ZIF‐8, MZM, and MZMU nanoparticle. D) SEM images of XDR‐PA after treated with PBS (control), US, MZMU, PMZMU, MZMU+US, and PMZMU+US. Scale bar：1 µm. E) Representative fluorescence images for live/dead bacterial staining assay of XDR‐PA after treated with PBS (control), US, MZMU, PMZMU, MZMU+US, and PMZMU+US, including PI signal (dead bacteria, red), SYTO‐9 signal (live bacteria, green), and merged pictures. Scale bar:200 µm.

**Figure 5:**
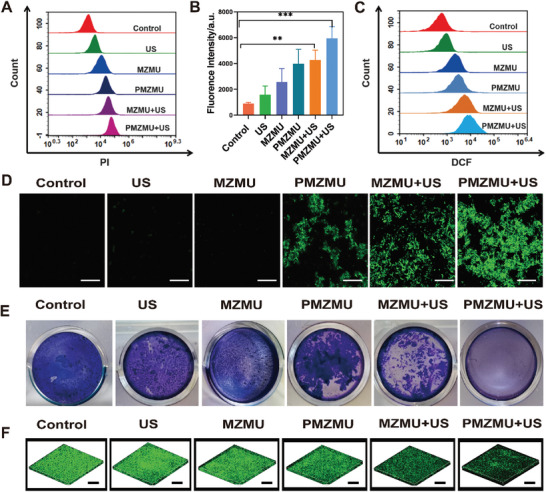
The antibacterial mechanism of PMZMU mediated sonodynamic therapy in vitro. A) Flow cytometry profiles of PI internalization by bacteria to evaluate membrane permeability after treated with PBS (Control), US, MZMU, PMZMU, MZMU+US and PMZMU+US. B) Quantitative analysis of PI uptake rate in bacteria after treated with PBS (Control), US, MZMU, PMZMU, MZMU+US and PMZMU+US. (n = 3, ^**^ *p* <0.01, ^***^ *p* <0.001. Data are expressed in mean ± SD). C) Flow cytometry profiles of ROS generation in XDR‐PA using flow cytometry with DCFH‐DA staining after treated with PBS (Control), US, MZMU, PMZMU, MZMU+US and PMZMU+US. D) Fluorescence images of XDR‐PA showing the production of ROS after different treatments. Scale bar:200 µm. E) Macroscopic XDR‐PA biofilm images of different treatment groups with crystal violet staining. F) 3D reconstructions of the fluorescence‐labeled XDR‐PA biofilms stained with SYTO‐9 (live bacteria) after different treatments. Scale bar:30 µm.

To assess the antibacterial activity of PMZMU in vitro, clinically isolated XDR *Pseudomonas aeruginosa* (XDR‐PA), *Acinetobacter baumannii* (XDR‐Ab), *Klebsiella pneumoniae* (XDR‐Kp) and *Escherichia coli* (XDR‐E. coli) were chosen as models for various treatments. To investigate whether the encapsulation of nanoparticles alters the antimicrobial efficacy of the drug, we determined the minimum inhibitory concentrations (MICs) of MZMU, PMZMU nanoparticles, and free PMB against four clinical bacterial isolates. As indicated in Table S1 (Supporting Information), MZMU did not exhibit inhibitory effects on the four bacterial strains. However, PMZMU demonstrated lower MIC values against XDR‐Kp, XDR‐Ab, and XDR‐PA than free PMB, while the MIC value against XDR‐*E. coli* remained similar to that of free PMB. These results suggested that nanoparticle encapsulation does not diminish the antibacterial effect of the loaded PMB. Therefore, in vitro and in vivo experiments were divided into the following groups: Control, US, MZMU, PMZMU, MZMU combined with ultrasound (MZMU+US), and PMZMU combined with ultrasound (PMZMU+US). Based on the standard colony counting method, PMZMU+US effectively combated all four XDR gram‐negative bacteria in vitro, while US and MZMU alone could not inhibit the growth of these bacteria. PMZMU+US gave inhibition rates of 86.5%, 71.0%, 85.7%, and 84.9% against XDR‐PA, XDR‐Ab, XDR‐Kp and XDR‐E. coli, respectively (**Figure** **4**A,B; Figure S11, Supporting Information). Given the remarkable antimicrobial effect of PMZMU+US against XDR‐PA, this bacterium was selected as the representative model for subsequent experiments. First, we used scanning electron microscope (SEM) to study the specificity of MZMU against XDR‐PA in vitro. As illustrated in Figure 4C, most nanoparticles in the MZM or MZMU groups attached to the surrounding and surface of the bacteria, while only a few in the TMOS@ZIF‐8 group attached, suggesting high specificity of MZMU for bacterial cells. Subsequently, we delved into the antimicrobial mechanisms of PMZMU‐mediated sonodynamic therapy. The change of bacterial morphology could be visually observed through SEM. The bacteria in the Control, US, and MZMU groups remained intact, but the shapes of the bacteria in the MZMU+US and PMZMU+US groups were significantly altered, and the wrinkling and lysis of the cell wall could be observed (Figure 4D). The dead/live stained laser confocal scanning microscope (CLSM) images revealed that almost all bacteria in the PMZMU+US group were dead (red fluorescence, PI, λex = 536 nm, λem = 617 nm), whereas the majority of bacterial cells in the other groups were alive (green fluorescence, SYTO‐9, λex = 488 nm, λem = 498 nm), suggesting that PMZMU+US had the highest destruction efficiency against XDR‐PA (Figure 4E). Quantitative analysis of PI fluorescence using flow cytometry also evidenced that SDT could significantly destroy the bacterial cell wall and membrane, presenting enhanced PI internalization into bacteria (**Figure** **5**A,B). To further investigate the mechanism responsible for bacteria disruption, 2′,7′‐dichlorofluorescein diacetate (DCFH‐DA), which can be oxidized by cytosolic ROS to produce strong green fluorescent 2′,7′‐dichlorofluorescein (DCF), was used to quantify the ROS formation. Flow cytometry and CLSM results showed the PMZMU+US group exhibited the most intense green fluorescence, indicating the highest ROS levels (Figure 5C,D; Figure S12, Supporting Information). Those results elucidated that the antibacterial activity of PMZMU‐mediated SDT was related to the formation of ROS, and ROS‐induced disruption of the cell wall and membrane was the potential killing mechanism of SDT.

### 2.6 Antibiofilm Activity

In the infectious microenvironment, bacteria protect themselves from the immune system and antibiotics by secreting extracellular polymeric substances (EPS) to form biofilms, thereby enhancing their resistance and leading to chronic infections and persistent diseases by blocking antibiotic infiltration.^[^ ^38^ ^]^ Therefore, we also investigated the efficacy of PMZMU mediated SDT in eradicating biofilm infections. Crystalline violet staining revealed that the biofilm retained high integrity following Control, US and MZMU groups, indicating that the dense structure of the biofilm was difficult to be disrupted by MZMU and US alone. Instead, MZMU+US and PMZMU+US disrupted the tight biofilm and caused significant biomass loss (Figure 5E). The quantitative results of crystalline violet staining exhibited that PMZMU+US group resulted in the lowest biofilm biomass (Figure S13, Supporting Information). Those results were also reflected in CLSM 3D reconstruction (Figure 5F).

### 2.7 Biodistribution and Pharmacokinetics of MZMU and PMB In Vivo

**Figure 6:**
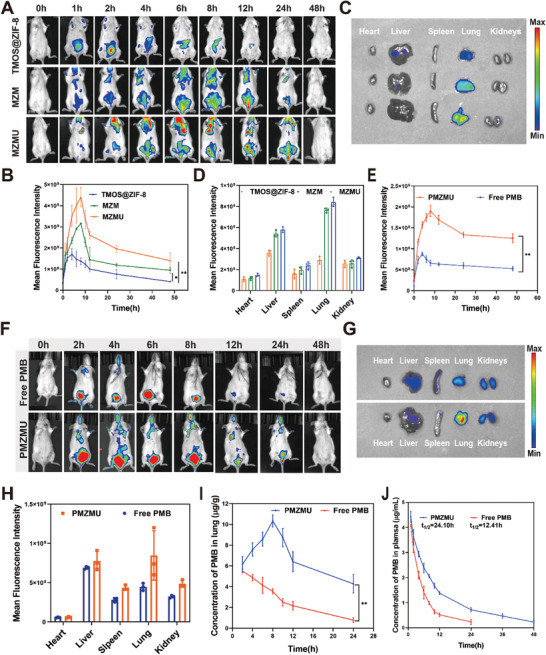
In vivo fluorescence imaging for the distribution of MZMU and PMB. A,B) In vivo fluorescence images of XDR‐PA lung infected mice and quantitative mean fluorescence intensity analysis of lung at different time points after intravenous injection with 100 µL TMOS@ZIF‐8, MZM and MZMU (4 mg mL^−1^). C,D) Ex vivo fluorescence images of major organs at 48 h after intravenous injection of TMOS@ZIF‐8, MZM and MZMU. E,F) In vivo biodistribution of PMB in XDR‐PA lung infected mice and quantitative mean fluorescence intensity analysis of lung at different time points after intravenous injection of free PMB and PMZMU (2 mg kg^−1^ PMB). G,H) Ex vivo fluorescence images of major organs at 48 h post‐injection of free PMB and PMZMU. I) The concentrations of PMB in lung tissues at comprehensive different time points. J) Pharmacokinetics of PMB in plasma. (n = 3, ^*^ *p* <0.05, ^**^ *p* <0.01, ^***^ *p* <0.001. Data are expressed in mean ± SD).

To compare the lung‐targeting ability of TMOS@ZIF‐8, MZM, and MZMU, we intravenously injected them into mice with XDR‐PA pneumonia, Then we monitored the fluorescence intensity of major organs in vivo and ex vivo at different time points using fluorescence imaging. As shown in **Figure** **6**A,B, TMOS@ZIF‐8 nanoparticles were quickly cleared in vivo due to their very short blood circulation half‐life, while MZM and MZMU exhibited much longer circulation in vivo and lightened the lungs at 1 h post‐injection. It was worth mentioning that the MZMU group exhibited the highest fluorescence intensity in the lungs, peaking at 8 h post‐injection, indicating that MZMU could accumulate in the lungs. The ex vivo imaging of major organs showed strong fluorescent signals in the lungs up to 48 h post‐injection of MZM and MZMU, compared to only weak fluorescence in the liver and lungs of the TMOS@ZIF‐8 group (Figure 6C,D). This confirmed the superior lung accumulation ability of MZMU due to prolonged circulation and the targeting effect of UBI_29‐41_ modified MSCm. Meanwhile, we visualized the distribution of PMB in pneumonia mice in vivo using Cy7‐NHS labeling of PMB. Further, we confirmed that PMZMU nanocarriers effectively targeted the delivery of PMB to lung tissue, which is crucial for enhancing the therapeutic effect of PMB (Figure 6E–H; Figure S14, Supporting Information). The quantitative analysis of PMB concentration in the lung tissue of the PMZMU group showed a 2.9‐fold and 5.5‐fold increase compared to the free PMB group at 8 h and 24 h respectively (Figure 6I). The plasma PMB concentration was measured after intravenous injection of free PMB or PMZMU using enzyme‐linked immunosorbent assay (ELISA) kits to explore the pharmacokinetics. Findings showed that the blood half‐life of free PMB was 12.41 h, while PMZMU's half‐life was 24.10 h, indicating a notable extension in circulation time (Figure 6J).

### 2.8 Antibacterial Effects of PMZMU + US In Vivo

**Figure 7:**
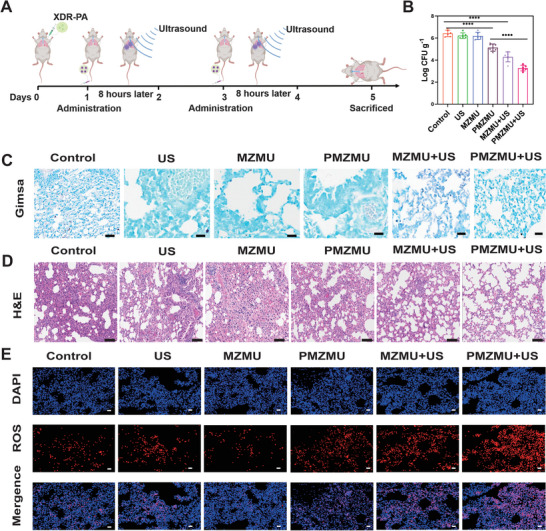
In vivo evaluation of antibacterial effects using the mouse acute XDR‐PA model. A) Schematic diagram of the in vivo treatment procedure for the antibacterial assay. B) Bacterial loads in lungs of mice after treated with saline (Control), US, MZMU, PMZMU, MZMU+US and PMZMU+US. (n = 6, ****p <0.0001,Data are expressed in mean ± SD). C) Giemsa staining of the lung tissue after various treatments. Scale bar:50 µm. D) H&E staining of the lung tissue after various treatments. Scale bar:50 µm. E) Fluorescence‐stained lung tissue sections in different treatment groups to measure ROS production in vivo. DAPI (blue fluorescence) represents the cell nucleus, while Dihydroethidium (red fluorescence) stands for ROS. Scale bar: 200 µm.

Motivated by the remarkable bactericidal effect observed in vitro, we previously evaluated the therapeutic potential of PMZMU+US in an acute XDR‐PA pneumonia mouse model. The treatment protocol of the PMZMU‐based treatment is illustrated in **Figure** **7**A. Briefly, mice were intratracheally inoculated with the XDR‐PA suspension and subsequently randomized into six groups based on their respective treatments: Control, US, MZMU, PMZMU, MZMU+US, and PMZMU+US. One day post‐infection, mice received intravenous injections of 100 µL of saline, MZMU, or PMZMU solutions (2 mg kg^−1^ PMB) according to their group assignment. Additionally, the US, MZMU+US, and PMZMU+US groups also underwent US irradiation 8 h after drug administration. Six mice per group infected with 100 µL of the XDR‐PA suspension (10^8^ CFU mL^−1^) were selected for survival and clinical scores monitoring, which was performed daily for seven days. As shown in Figure S15 (Supporting Information), mice treated with PMZMU+US exhibited a significantly higher survival rate (66.7%) and higher clinical symptom scores (Figure S16, Supporting Information) compared to other treatment groups. An additional thirty‐six mice were prepared for subsequent antibacterial experiments by infecting with 100 µL of the XDR‐PA suspension (5 × 10^7^ CFU mL^−1^). After completing two rounds of treatment, the mice were sacrificed, and their lungs were harvested for bacterial burden assessment. As indicated in Figure 7B, the bacterial burden in the lungs was significantly reduced in the PMZMU+US group, indicating the most effective bactericidal activity. Notably, we observed a substantial decrease in bacterial burden in the lungs of PMZMU‐treated mice compared to free PMB‐treated mice (Figure S17, Supporting Information), indicating that PMZMU possessed superior bactericidal capability over free PMB. This enhanced effect might be attributed to the nanoparticles' ability to improve drug concentration in lung tissues. Giemsa staining further confirmed the minimal bacterial residue in lung tissue of PMZMU+US group (Figure 7C). These results suggest that PMZMU+US exhibited the strongest bactericidal activity and effectively eliminated XDR‐PA in lung tissues.

### 2.9 Anti‐Inflammatory Effects of PMZMU + US In Vivo

We further assessed lung inflammation and cytokine generation in mice with different treatments to illustrate the beneficial effects of PMZMU on treating acute XDR‐PA pneumonia. Mice treated with PMZMU + US had the slightest inflammation in the lungs, showing the mildest pneumorrhagia (Figure S18, Supporting Information) and pulmonary edema as evidenced by the lower wet‐to‐dry ratio of lungs, compared with the other five groups (Figure S19, Supporting Information). In addition, the histological analysis revealed that lungs from control mice exhibited severe alveolar structure damage and massive infiltration of inflammatory cells, whereas lungs from mice treated with PMZMU + US retained alveolar integrity and empty space, essential for efficient gas exchange (Figure 7D). Pro‐inflammatory cytokines, including TNF‐α and IL‐6, as well as anti‐inflammatory cytokines (IL‐10) in the serum of mice were detected by ELISA. TNF‐α and IL‐6 levels were lower, while IL‐10 levels were higher in the PMZMU + US group compared to all other treatment groups (Figure S20, Supporting Information). The findings revealed that PMZMU+US treatment mitigated pulmonary edema and congestion, and reduced levels of pro‐inflammatory cytokines, which was crucial for protecting mice from lung injury caused by excessive inflammatory response. Moreover, to validate the therapeutic mechanism in vivo, we employed DAPI and dihydroethidium staining to assess ROS production in the lungs of mice across different treatment groups (Figure 7E). Notably, the PMZMU + US cohort exhibited the highest levels of red fluorescence signals, indicating that PMZMU can be effectively activated by US to generate ROS and enhance the antimicrobial effect of SDT upon delivery to the lungs.

### 2.10 In Vitro Immunomodulatory Function of PMZMU

**Figure 8:**
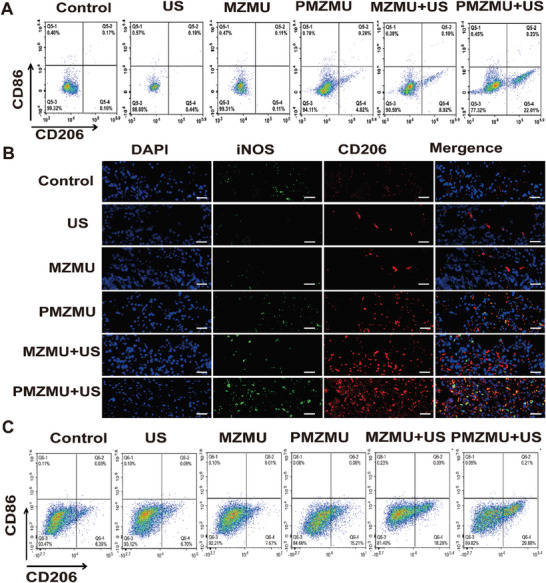
In vitro and in vivo evaluation of immunomodulatory functions. A) Flow scatter diagrams of CD86 (M_1_ marker) and CD206 (M_2_ marker) expression in macrophages (RAW 264.7) cells after different treatments B) Immunofluorescent co‐staining of CD206/iNOS/DAPI in lung tissues. Scale bar:50 µm. C) Flow cytometry graphs of the percentage of M_2_ macrophages (CD45^+^CD11b^+^F4/80^+^CD206^+^) and M_1_ macrophages (CD45^+^CD11b^+^F4/80^+^CD86^+^) in the spleen of mice in different treatment groups (n = 6）.

Macrophages play a crucial role in defending the body against infection by recognizing, phagocytizing, and eliminating bacteria. These versatile cells polarize in response to various stimuli and environmental cues, exhibiting distinct functions in antibacterial and anti‐inflammatory processes. Multiple studies have found that SDT effectively stimulates macrophage polarization toward the M_2_ phenotype, significantly reducing localized inflammation. Mechanistically, nonlethal sonodynamic therapy (NL‐SDT) stimulates macrophage M_2_ polarization by activating the ROS‐AMPK‐mTORC1‐autophagy signaling pathway in murine bone marrow‐derived M_1_ macrophages.^[^ ^39^ ^]^ Accordingly, macrophage RAW264.7 was co‐cultured with MZMU or PMZMU for 24 h, and then examined by flow cytometry. The flow scatter diagrams showed amplified CD206‐positive cells (M_2_) in the PMZMU, MZMU + US and PMZMU + US groups, with no significant change in the MZMU group (**Figure** **8**A; Figure S21A, Supporting Information). However, the number of CD86‐positive cells were unperturbed (Figure S21B, Supporting Information). This indicates that PMZMU‐mediated SDT promotes M_2_ polarization.

### 2.11 In Vivo Immunomodulation

Immunofluorescence staining for CD206/nitric oxide synthase (iNOS) showed more CD206‐positive staining in the PMZMU +US group, suggesting upregulation of the number of M_2_ macrophages (Figure 8B). After utilizing flow cytometry to examine the quantity of CD206‐labeled M_2_ macrophages in the spleens of mice, our findings indicate that the proportion of M_2_ cells was elevated in the PMZMU, PMZMU+US, and PMZMU+US groups when compared to the control, US, and MZMU groups (Figure 8C). Notably, the PMZMU+US group exhibited the highest count of M_2_ cells (Figure S22, Supporting Information). These data underpin the superior efficacy of PMZMU+US in modulating macrophage polarization toward the M_2_ phenotype, thereby mitigating pathological damage to lung tissues caused by inflammation (Figure 8D).

### 2.12 Evaluation of the Biocompatibility of PMZMU

The airway epithelium is a significant line of defense against various inhalation challenges and plays a crucial role in host immunity.^[^ ^40^ ^]^ The human bronchial epithelial cell (BEAS‐2B) cell line, derived from normal human bronchial epithelial cells, is able to mimic the response of lung epithelial cells under physiological and pathological conditions, and has become an essential tool in the study of lung diseases and the assessment of drug toxicity.^[^ ^41^, ^42^ ^]^ Therefore, we chose BEAS‐2B for MTT assay to evaluate the cytotoxicity of PMZMU nanoparticles. As shown in Figure S23 (Supporting Information), PMZMU exhibited good biocompatibility, with cell survival rates higher than 80% in all groups, even at concentrations up to 100 µg mL^−1^. Similar results were obtained in the hemolysis assay (Figure S24, Supporting Information). Therefore, the concentration of PMZMU (100 µg mL^−1^) was finalized as the optimal dose for all in vitro experiments. The potential long‐term adverse effects of US, MZMU, PMZMU, MZMU+US, and PMZMU+US were evaluated in healthy mice. During the 28‐day observation period, no mortality was observed in any of the mice (Figure S25, Supporting Information). However, mice treated with PMZMU and PMZMU+US showed decreased body weight in the first three days after iv administration. In contrast, there was no significant change in body weight between US, MZMU, MZMU+US and saline‐treated mice (Figure S26, Supporting Information). The effects of PMZMU and PMZMU+US on body weight may be attributed to the leakage of PMB during circulation, which impairs appetite or intestinal absorption in mice. The counts of white blood cells (WBC), red blood cells (RBC), platelets (PLT) (Figure S27, Supporting Information), and the level of glutamic pyruvic transaminase (ALT), aspartate aminotransferase (AST), and creatinine were all in the normal range (Figure S28, Supporting Information), indicating that MZMU, PMZMU and US had no adverse effect on the hematopoietic system, liver, and kidney function. Moreover, no detectable pathological changes or adverse effects were observed in the H&E staining of major organs, further validating the favorable biosafety of PMZMU nanoparticles and its mediated SDT (Figure S29, Supporting Information).

## 3 Conclusion

In conclusion, our study demonstrates the remarkable potential of nanoengineered human umbilical cord mesenchymal stem cells as a sonosensitizer for synergistic sonodynamic‐nano‐antimicrobial therapy against gram‐negative XDR bacteria. The nanocarriers can precisely deliver antibacterial agents and sonosensitizers to infectious lung tissues, thereby enhancing drug distribution and initiating antibacterial sonodynamic therapy under ultrasound irrigation. Treated with PMZMU combined with ultrasound, survival rates significantly improved, bacterial counts decreased, and inflammatory damage was mitigated by regulating the M_2_ polarization of macrophages. Therefore, the integration of antibacterial nanoplatform and sonodynamic therapy may represent a potential approach for the treatment of XDR‐bacteria‐infected lung diseases, with minimal systemic toxicity.

## 4 Experimental Section

### Materials

Cetyltrimethylammonium chloride (CTAC) solution (25 wt.% in H2O) was purchased from Sigma‐Aldrich. Polymyxin B (PMB) was purchased from ShangHai YuanYe Biotechnology Co., Ltd. (USP Grade, 6000 IU mg^−1^). Tetraethyl orthosilicate (TEOS), bis[3‐(triethoxysilyl) propyl] tetrasulfide (BTES), ammonium hydroxide, triethanolamine (TEA),fluorescein 5‐isothiocyanate (FITC), (3‐Aminopropyl) triethoxysilane (APTES), Meso‐tetra(4‐car‐boxyphenyl) porphine (TCPP),2′,7′‐dichlorofluorescein diacetate (DCFH‐DA), 5,5′‐Dithiobis(2‐nitrobenzoic acid) (DTNB),glutathione(GSH),N‐Hydroxysuccinimide(NHS), 1‐ethyl‐3‐(3‐(dimethylamino) propyl) carbodimide (EDC),N,N‐Dimethylformamide(DMF),zinc nitrate hexahydrate (Zn(NO_3_)_2_·6H_2_O), and 2‐methylimidazole (2‐MeIm) were purchased from Aladdin Reagent Co., Ltd. (Shanghai, China). All reagents were of analytical grade and used without further purification. Polymyxin B ELISA kit (EK‐M21417) was purchased from EK‐Bioscience. Mouse cytokine (IL‐6, IL‐10 and TNF‐α) ELISA kits were purchased from AiFang Biological Co., Ltd. SYTO‐9/PI Live and Dead Bacteria Stain Kit was purchased from Zeye Co., Ltd. (Shanghai, China). DSPE‐PEG_2000_‐Mal (Y5‐D22205) and Sulfo‐Cyanine 7 NHS ester (477908‐53‐5) were purchased from Chongqing Yusi Pharmaceutical Technology Co., Ltd. (Chongqing, China). Antibodies to PerCP‐CD45(103 130), PE/cy7‐CD11b (101 215), PE‐CD86(159 204), APC‐F4/80(123 116), and FITC‐CD206 (141 704) were purchased from BioLegend. Fixable EF450(65‐0863) was purchased from Invitrogen.

### Preparation of TMOS

CTAC (2 g) and TEA (0.1 g) were dissolved in 20 mL of ultrapure water and stirred for 1 h. After transferring the mixture to an oil bath maintained at 80 °C, 1 mL of TEOS was added and reacted for 1 h again. Subsequently, a mixture of 2 mL of BTES and 1 mL of TEOS was added and reacted for 2 h to obtain MOS cores. TCPP‐silane (1 mL) was added to the MOS solution and reacted for 2 h to obtain TMOS. Then the core/shell structured TMOS product was collected by centrifugation and washed with pure ethanol three times. Finally, the residual CTAC residue was extracted with NaCl in 30 mL of methanol, and the resultant CTAC‐free TMOS products were dissolved in ultrapure water. TCPP‐silane was synthesized through amidation of TCPP with APTES in DMF the day before the above experiment. The steps were as follows:0.126 mmol TCPP, 0.164 mmol APTES, 0.378 mmol NHS and 0.378 mmol EDC were dissolved in 2.5 mL DMF solution and stirred overnight at room temperature.

### Preparation of TMOS@ZIF‐8

400 µL TMOS solution (10 mg mL^−1^) was mixed with 80 µL Zn (NO_3_)_2_·6H_2_O (61.58 mg mL^−1^) and transferred to an ice bath for 15 min of sonication. Afterward, 800 µL 2‐MeIm (77.10 mg mL^−1^) were quickly added to the above mixing system for another 15 min of sonication in an ice bath. Finally, the synthesized TMOS@ZIF‐8 products were collected by centrifugation and washed four times with ethanol.

### Preparation of MSC Membrane and TMOS@ZIF‐8@MSCm (MZM)

The primary human umbilical cord mesenchymal stem cells (MSCs) were donated by the Jiangsu Cell Tech Medical Research Institute and cultured in DMEM containing 10% FBS. When third‐generation MSC cells in the T75 flask reached 80 to 90% confluence, they were washed three times with ice‐cold PBS buffer and then harvested using 0.25% Trypsin‐Ethylene Diamine Tetraacetic Acid (EDTA). After being washed three times, the MSCs were resuspended in hypotonic solution and lysed overnight at 4 °C. The lysed cells were pulverized in an ultrasonic cell crusher for 5 min and centrifuged at 3500 rpm for 10 min to remove the cellular contents. Subsequently, centrifugation was repeated at 4 °C and 14800 rpm for 2 h, and the precipitate was collected as the MSC membranes (MSCm). Finally, the purified MSCm from 1 × 10^8^ cells were mixed with TMOS@ZIF‐8 (1 mL, 1 mg mL^−1^) and sequentially extruded through a polycarbonate membrane with pore sizes of 400,200,and 100 nm using an Avanti Mini‐Extruder to form TMOS@ZIF‐8@MSCm (MZM)

### Preparation of TMOS@ZIF‐8@MSCm‐UBI_29‐41_(MZMU)

UBI_29‐41_, labeled with FITC‐Ahx at the N‐terminus and cysteine at the C‐terminus, was produced by Nanjing Kingsley Co., Ltd. The final amino acid sequence was TGRAKRRMQYNRRC. The molecular weight of UBI_29‐41_ was 2298.64 Da, as confirmed by mass spectrometry (Figure S30A, Supporting Information). The purity of the synthesized peptide was ≥98.0%, as determined by High‐ Performance Liquid Chromatography (HPLC) (Figure S30B, Supporting Information). UBI_29‐41_ (1 mg) was added to 5 mL of Mal‐PEG_2000_‐DSPE solution (2 mg mL^−1^), stirred for 6 h, and then purified using a dialysis bag (MWCO 3500 Da), to finally obtain DSPE‐PEG_2000_‐UBI_29‐41_. Then DSPE‐PEG_2000_‐UBI_29‐41_ was added to TMOS@ZIF‐8@MSCm solution and stirred for 2 h to form MZMU nanoparticles. MSC membrane proteins were analyzed using sodium dodecyl sulfate‐polyacrylamide gel electrophoresis (SDS‐PAGE). Briefly, the protein concentrations of whole MSCs, MSCs membranes, TMOS@ZIF‐8 and MZMU were quantified with the Bicinchoninic Acid Assay kit. After being denatured, each specimen (20 µg) was added into a 10% SDS‐PAGE, ran at 80 V for 2 h, and then stained with Coomassie blue. Subsequently, the gel was washed by deionized water, decolorized with decolorizing solution and imaged with camera.

### Preparation of PMB‐Loaded MZMU(PMZMU)

TMOS (10 mg) and PMB (20 mg) were dissolved in 10 mL of PBS and stirred at 4 °C for 24 h to obtain PMB@TMOS. Excessive unloaded PMB in the mixture was removed by centrifugation and washed with PBS three times. PMB@TMOS was then sequentially coated with ZIF‐8 and MSCm‐UBI_29‐41_ to obtain PMB‐loaded MZMU (PMZMU). Thermogravimetric analysis (TGA) was performed to determine the loading amount of PMB in PMZMU under argon and oxygen atmospheres, with a heating rate of 10 °C min^−1^ from 30 to 800 °C.

### Characterization

The morphology of the nanoparticles was characterized using a transmission electron microscope (TEM). Dynamic light scattering (DLS, 25 °C) and zeta potentials of the nanoparticles were determined by DLS (Malvern Instruments, UK). BET surface area and pore size distribution were analyzed using N_2_ adsorption‐desorption isotherms (Tristar II 3020 M). The elemental compositions of TMOS and TMOS@ZIF‐8 were examined using element‐mapping analysis. X‐ray photoelectron spectroscopy (0–1400 eV), energy‐dispersive X‐ray spectroscopy (EDS), and X‐ray diffraction (XRD) were employed to investigate the structure of nanoparticles (scan angle: 5°−40°, scan speed: 4° min^−1^). Electron paramagnetic resonance (EPR) spectrometry (Bruker, Germany) was used to detect ^1^O2 generation. The modification of UBI_29‐41_ on MSCm was verified by fourier transform infrared spectroscopy (FTIR) and UV‐Vis Spectrophotometer (Wavelength 300–600 nm).

### Macrophage Uptake of MZMU

RAW264.7 cells were seeded in a confocal dish (3 × 10^4^ cells/dish) and cultured overnight. TMOS@ZIF‐8 and MZMU were incubated with cells at a final NP concentration of 25 µg mL ^−1^. After 1 or 6 h of incubation, cells were stained with DAPI for observation by CLSM.

### Colocalization

To demonstrate the co‐localization of the MSC membranes and the TMOS@ZIF‐8 cores, RAW264.7 cells were seeded in a confocal dish (3 × 10^4^ cells/dish) and cultured overnight. Cells were incubated with TMOS@ZIF‐8@MSCm‐DiO at a final NP concentration of 25 µg mL ^−1^. After 2 h of incubation, cells were stained with DAPI and then observed by CLSM.

### Determination of Singlet Oxygen Generation

The production singlet oxygen from MZMU was examined by monitoring the fluorescence decrease of ABDA, a singlet oxygen sensor. In brief, 100 µL of the ABDA solution (100 µg mL^−1^) was carefully mixed with 900 µL of MZMU suspension containing 100 µg of MZMU nanoparticles. The mixture was then exposed to different durations of ultrasound (1.5 W cm^−2^, 1.0 MHz, 50% duty cycle). Subsequently, the absorption of ABDA at 400 nm at different time points was recorded using a UV spectrophotometer.

### PMB Release from PMB@TMOS, PMB@TMOS@ZIF‐8, and PMZMU

The in vitro release of PMB from PMB@TMOS, PMB@TMOS@ZIF‐8, and PMZMU were evaluated using a dialysis bag diffusion method. In brief, 10 mg of PMB@TMOS, PMB@TMOS@ZIF‐8 and PMZMU in 2 mL of PBS was transferred into a pre‐processed dialysis membrane (3500 MWCO), followed by immersion into 18 mL of PBS at different pH values (7.4,6.5,5.5, and 5.5+5 mm GSH). The dialysis was conducted at 37 °C in a shaking culture incubator (300 rpm). At the indicated time points of 0, 0.5, 1, 2, 4, 6, 8, 12, 18, 24, and 48 h, 1 mL aliquots of sample solutions were removed from the incubation medium for measurement and compensated by adding 1 mL of fresh buffer to the incubation medium. The temporal evolution of PMB content in the dialysate was monitored with PMB ELISA kit.

### TCPP Release from PMZMU

For the TCPP release test, PMZMU was dispersed in 2 mL pH 5.5 PBS containing GSH (5 mm) and gently shaken at 100 rpm at 37 °C. At predetermined time points (0, 0.5,1,2,4,6,8,10,12,24,48,72, and 168 h), the amount of released GSH in the supernatant was measured. The released amount of TCPP was quantified by UV–vis spectrophotometer (Wavelength 425 nm).

### Zinc Ions Release from PMZMU

In vitro, release of Zinc ions from PMZMU was measured according to the above procedures. The concentrations of Zinc ions in the released solutions were quantified by ICP‐MS measurement. Briefly, the standard solutions of Zinc ions were sequentially diluted to different concentrations using 2% nitric acid, then measured by ICP‐MS to plot the standard curve. Subsequently, 0.5 mL of the sample was taken and 5 mL of superior grade nitric acid was added for microwave digestion. After digestion, the sample was cooled and diluted with 2% nitric acid to achieve concentrations of 25 ppb, 50 ppb, and 100 ppb by transferring 5, 10, and 20 mL of the diluted sample, respectively, into 100 mL volumetric flasks. Finally, the content of Zinc ions in the samples was determined by ICP‐MS (Limit of Quantitation:0.0030ppb).

### In Vitro Cytotoxicity

The cytotoxicity of MZMU and PMZMU were determined by MTT assay. BEAS‐2B cells in logarithmic growth phase were co‐cultured with different concentrations of nanoparticles (10,25,50, 100, 125, 250, 500, 1000, and 1500 µg mL^−1^) for 24 h. The control group added an equal volume of sterilized PBS. Relative cell viability was calculated as the percentage in relation to untreated control cells.

### In Vitro Antibacterial Activity Assay

Four clinically isolated XDR strains (XDR‐PA, XDR‐Ab, XDR‐Kp, and XDR‐E. coli) were kindly provided by the Department of Laboratory Medicine, The Second Affiliated Hospital of Nanjing Medical University for the experiments. Ethical approval for using these bacteria strains was granted by the Human Ethics Committee of the Second Affiliated Hospital of Nanjing Medical University (Approval Number: 2024‐KY‐165‐01). The drug sensitivity of these bacterial strains is shown in Table S2 (Supporting Information). The details about the source patients are listed in Table S3 (Supporting Information). Bacterial strains were grown in lysogeny broth (LB) medium under shaking conditions at 37 °C until they reached the logarithmic growth phase before proceeding with the experiments. The concentration of the first‐generation bacteria was determined by measuring the optical density at 600 nm (OD600) using UV–vis spectroscopy. Bacteria suspensions were divided into six groups: Control, US, MZMU, PMZMU, MZMU +US, and PMZMU +US. The plate counting method was utilized to assess the antibacterial efficacy of PMZMU against bacteria in vitro. The final concentrations of bacteria and PMZMU were 10^6^ CFU mL^−1^ and 100 µg mL^−1^, respectively. An equal volume of PBS was added to the control group. The experimental conditions for ultrasound exposure were set at 1.5 W cm^−2^, 1.0 MHz and 50% duty cycle for 8 min. After different treatment as groups, 100 µL of suspensions were withdrawn and serially diluted. Subsequently, 100 µL of the diluted bacterial solutions were plated onto LB agar plates and incubated in a bacterial incubator at 37 °C. After being incubated for 18 h, the complete and not fused bacterial colonies were enumerated. Experiment was repeated at least three times.

### Determination of Minimum Inhibitory Concentrations (MIC)

Four clinically isolated XDR strains were cultured in LB media at 37 °C with rotation (180 rpm) overnight. MIC for antimicrobial susceptibility testing of MZMU, PMZMU nanoparticles and PMB were performed through the broth dilution method according to the guidelines of the Clinical and Laboratory Standards Institute. The bacterial concentration was adjusted to 10^5^ CFU mL^−1^. 100 µL of bacterial culture was added to a 96‐well plate and incubated (37 °C) with 100 µL MZMU, PMZMU nanoparticles or PMB solutions at different concentrations in 96‐well plates. After 24 h of incubation, the MIC value was determined by evaluating the visible growth of microorganisms.

### Live/Dead Fluorescent Staining

Bacterial cultures were washed twice with PBS, centrifuged (6000 rpm,5 min), and then serially diluted. The bacteria suspensions (10^6^ CFU mL^−1^) incubated with 100 µL MZMU and PMZMU before and after ultrasound irradiation were treated with the mixture of SYTO 9 and PI for 30 min in the dark. After washed three times with PBS, the treated bacterial suspension was transferred to a confocal petri dish and observed by laser confocal scanning microscope (CLSM). The experiments performed with PBS treatment were used as control groups.

### Flow Cytometry Analysis (FCM) for Membrane Permeability Measurement

After different treatment as groups, the bacteria were harvested and incubated with PI (10 µg mL^−1^) for 30 min in the dark and then observed immediately using FCM with the excitation setting at 488 nm.

### Inhibition of Bacterial Layer Formation

The experiment was divided into six groups as before, MZMU and PMZMU nanoparticles (100 µL) were added to 100 µL of bacterial suspension (1 × 10^7^ CFU mL^−1^). Control and US groups were added 100 µL of PBS. After 4 h of co‐culture, US, MZMU+US, and PMZMU+US groups were given ultrasound irradiation. After continuing the incubation for 24 h, the wells were washed three times with PBS and then incubated for 1 h by adding 0.1% (v/v) crystal violet solution (200 µL mL^−1^). Then, 200 µL of 95% (v/v) ethanol was added to dissolve the crystal violet. The experiment was repeated three times and the absorbance was measured at 595 nm (microplate reader, Synergy NEO, BioTek, USA). The inhibition rate of bacterial layer formation was calculated according to the following formula:

(1) $$\begin{array}{ccc} & & \mathrm{Inhibition}\;\mathrm{rate}\;\mathrm{of}\;\mathrm{bacterial}\;\mathrm{layer}\;\mathrm{formation}\;\left(\%\right)=\left[1-\left(\mathrm{absorbance}\;\mathrm{of}\;\mathrm{drug}\right.\right.\\ & & \left.\left.-\;\mathrm{treated}\;\mathrm{cells}/\mathrm{absorbance}\;\mathrm{of}\;\mathrm{untreated}\;\mathrm{control}\;\mathrm{cells}\right)\right]\times 100 \end{array}$$

### SEM Observation

Bacteria treated as described above were washed three times with PBS and fixed with phosphate buffer containing 2.5% glutaraldehyde for 2 h. The suspension was then dehydrated with graded ethanol (30%, 50%, 70%, 90%, 100%) for 10 min. A coverslip was used to spread the mixture and allowed to dry naturally. Finally, the coverslips were sputter‐coated with gold to observe SEM images.

### Intracellular ROS Measurement

Bacteria suspensions, grouped as above, were incubated with PBS, MZMU, and PMZMU in the dark for 4 h respectively. After rinsing, the bacterial pellets were resuspended in PBS and treated with ultrasound irradiations. Afterward, the bacterial cells were immediately harvested and stained with 100 µL 2,7‐dichlorodihydrouorescein diacetate (DCFH‐DA,10 µm) for 30 min in the dark and finally washed with PBS. Subsequently, the bacteria were observed using CLSM, and the relative fluorescence intensity within the cells was measured by flow cytometry, with an excitation wavelength set at 488 nm.

### FCM for Macrophage Polarization

RAW264.7 cells were inoculated into 12‐well plates at a density of 10^5^ cells well^−1^. After 12 h, 1 mL of DMEM containing nanoparticles (50 µg) was added to each well, while an equal volume of DMEM without nanoparticles was added to the control group. After incubation for 24 h, RAW 264.7 cells were collected and resuspended in 100 µL of PBS containing (FITC)‐labeled CD206 antibody (2.5 µL) and P‐phycoerythrin (PE)‐labeled CD86 antibody (2.5 µL), and sequentially incubated in an ice bath for 30 min. Then, residual antibody was removed and the cells were re‐suspended in 400 µL PBS. Finally, the CD86 and CD206 expression in RAW264.7 cells was detected using FCM. Three replicates were conducted for each group.

### Animal Studies—Animal experiment ethics

Female BALB/c mice (6‐8 weeks old, 18–20 g) were purchased from Yangzhou University Laboratory Animal Center for in vivo experiments. All animal experiments were approved by the Institutional Animal Care and Use Committee (Approval Number: 2404092). All handling and surgical procedures followed the approved guidelines

### Animal Studies—In Vivo Biodistribution of MZMU

To minimize the damage to the mice, an acute pneumonia model in mice was established by airway perfusion. Briefly, after intraperitoneal anesthesia with 2% pentobarbital sodium, the nasal cavity of the mice was pinched tightly with forceps, and then 100 µL of bacterial suspension was instilled into the airway of the mice using a gavage needle. The mice were shaken vertically for 1 min to ensure that the bacterial solution entered the lungs of the mice. Pneumonia mice were divided into three different groups (n = 3, per group) to investigate the biodistribution of TMOS@ZIF‐8, TMOS@ZIF‐8@MSCm, and MZMU. Since the sonosensitizer TCPP contained in the nanoparticles can be used for in vivo imaging of animals, the nanoparticles were not labeled with additional dyes. Mice were intravenously administrated with TMOS@ZIF‐8, TMOS@ZIF‐8@MSCm (MZM), and MZMU solution (0.4 mg mL^−1^ TCPP) respectively, and imaged at designed time points (1, 2, 4, 6, 8, 12, 24, and 48 h). At 48 h post‐injection, major organs collected from the executed mice were imaged on the IVIS imaging system.

### In Vivo Antibacterial Study

Female BALB/c mice (6 to 8 weeks old) were randomly divided into six groups: Control, US, MZMU, PMZMU, MZMU+US, and PMZMU+US. 100 µL of bacterial suspensions (10^8^ CFU mL^−1^) were administered intratracheally to induce severe acute pneumonia for monitoring survival and clinical symptom scores (n = 6, per group). To evaluate the antimicrobial efficacy of PMZMU, additional mice were intratracheally inoculated with 100 µL of PBS containing 5 × 10^6^ CFU of XDR‐PA. After 24 h, saline, MZMU, and PMZMU (2 mg kg^−1^ PMB) were injected into the tail veins of the mice according to the grouping. US (1.5 W cm^−2^, 1.0 MHz, 50% duty cycle, 8 min) was applied 8 h after the tail vein injection in the US, MZMU+US, and PMZMU+US groups. This treatment was repeated on the third day, and the mice were sacrificed on the fifth day. Subsequently, blood and lung tissue samples were collected to measure IL‐6, IL‐10, and TNF‐α levels by ELISA and to determine bacterial loads.

### Pharmacokinetic Study of PMZMU

Mice were intravenously injected with 100 µL of free PMB and PMZMU (2 mg kg^−1^ of equivalent PMB, n = 3 per group). At 1, 2, 4, 6, 8, 12, 24, 36, and 48 h after injection, 100 µL of blood was collected from the orbital vein of mice and dissolved in 900 µL of saline containing 10 mm EDTA anticoagulant. The plasma drug concentration of PMB was measured using an ELISA kit and a blood terminal half‐life curve was fitted according to a single‐compartment pharmacokinetic model.

### Immunofluorescence Staining

Lung tissues were collected and immersed in paraformaldehyde solution. After dehydration, the samples were embedded in paraffin and sections were co‐stained for DAPI/iNOS/CD206 immunofluorescence. All sections were observed under a fluorescence microscope.

### Flow Cytometry Analysis

Single‐spleen cell suspensions were obtained from sacrificed mice (n = 6). Cells (1 × 10^6^ mL^−1^) were blocked with Fc blocking antibody (BD Bioscience) for 15 min on ice. Cells were washed and stained with fluorochrome‐conjugated antibodies against macrophage surface receptors PerCP‐CD45, PE/cy7‐CD11b, PE‐CD86, APC‐F4/80, and FITC‐CD206 for 30 min on ice. Excess antibody was removed with PBS and the cells were resuspended in 400 µL PBS. Data were collected on a flow cytometer and analyzed using Flow Jo software (Tree Star Inc, Ashland, OR, USA).

### Hemolysis Test of PMZMU

Red blood cells (RBC) were isolated from fresh mouse blood and centrifuged at 400 g for 10 min. Cells were purified by rinsing five times with saline. The RBC suspension was diluted 10‐fold in saline and 0.1 mL of RBC suspension was added to 0.9 mL of water (positive control), saline (negative control), or saline containing various concentrations of PMZMU. After incubation at 37 °C for 2 h, the supernatant was collected and the OD value at 540 nm was measured using an enzyme marker. The percentage of hemolysis was calculated using the following formula:

(2) $$\begin{array}{ccc} \mathrm{Hemolysis}\;\left(\%\right) & = & \left(\mathrm{OD}\;\mathrm{sample}-\mathrm{OD}\;\mathrm{negative}\right)/\\ & & \left(\mathrm{OD}\;\mathrm{positive}-\mathrm{OD}\;\mathrm{negative}\right)\times 100 \end{array}$$

### Statistical Analysis

All the experiments were performed at least three times. Statistical analysis was conducted with GraphPad Prism 8 software. Data were expressed as mean ± standard deviation (SD) and sample size (n) for each statistical analysis was represented in the corresponding figure legends. Differences between groups were analyzed using one‐way analysis of variance (ANOVA) followed by Tukey's multiple comparisons test. A comparison between two groups was performed using the Student's t‐test. P value of less than 0.05 was considered significant. ^*^ *p* <0.05, ^**^ *p* <0.01, and ^***^ *p* <0.001.

## Conflict of Interest

The authors declare no conflict of interest.

## Author Contributions

J.L.H., S.X.C., and X.W.H. designed and synthesized the materials. J.L.H., X.J., H.H.Y., Y.C.H., and F.L.G. conducted the material characterizations. J.L.H., C.H.Z., and M.Y.S. Y.X.Y. conducted and analyzed most of the in vitro and in vivo experiments. X.R.D. and G.Z.F. provided important experimental insights and cowrote the paper. All authors discussed, commented, and agreed on the manuscript.

## Supporting information

Supporting Information

## Data Availability

The data that support the findings of this study are available from the corresponding author upon reasonable request.
