# Supplementary material for: Biomimetic Metal–Organic Framework Gated Nanoplatform for Sonodynamic Therapy against Extensively Drug Resistant Bacterial Lung Infection
Source: Adv Sci (Weinh). 2024 Jul 4;11(33):2402473. doi: 10.1002/advs.202402473 (PMC11434100; doi:10.1002/advs.202402473)
Supplement: Supplementary file 1 — Supporting Information [file ADVS-11-2402473-s001.pdf]

ADVANCED SCIENCE
Open Access

# Supporting Information

for *Adv. Sci.*, DOI 10.1002/advs.202402473

Biomimetic Metal–Organic Framework Gated Nanoplatform for Sonodynamic Therapy against Extensively Drug Resistant Bacterial Lung Infection

*Jianling Huang, Xiuwen Hong, Sixi Chen, Yucong He, Lixu Xie, Fenglin Gao, Chenghua Zhu, Xiao Jin, Haihao Yan, Yongxia Ye, Mingyue Shao, Xingran Du* and Ganzhu Feng**

Supporting information for

# Biomimetic Metal Organic Framework Gated Nanoplatform for Sonodynamic Therapy against Extensively Drug Resistant Bacterial Lung Infection

*Jianling Huang[1], Xiuwen Hong[1], Sixi Chen[1], Yucong He[1], Lixu Xie[2], Fenglin Gao[1], Chenghua Zhu[1], Xiao Jin[1], Haihao Yan[1], Yongxia Ye[3], Mingyue Shao[1], Xingran Du[4]*, Ganzhu Feng[1]**

Jianling Huang *et al.*

*Corresponding author. Email: xingrandu@njmu.edu.cn(X.R.D)

fenggz@njum.edu.cn (G.Z.F)

**Abbreviation and description of different preparations.**

| Abbreviation | Description |
|---|---|
| CM-NPs | Cell membrane-coated nanoparticles |
| hUC-MSCs | Human umbilical cord mesenchymal stem cells |
| XDR | Extensively drug-resistant |
| MSCm | Human umbilical cord mesenchymal stem cells membrane |
| MZM | MSCm coated nanoparticle of zeolite imidazole framework-8 sealed TCPP-doped mesoporous organo-silica |
| MZMU | $UBI_{29-41}$ modified MSCm coated nanoparticle of zeolite imidazole framework-8 sealed TCPP-doped mesoporous organo-silica |
| MOS | Mesoporous organo-silica |
| MOF | Metal-organic framework |
| PMB | Polymyxin B |
| PMZMU | $UBI_{29-41}$ modified MSCm coated nanoparticle of zeolite imidazole framework-8 sealed TCPP-doped mesoporous organo-silica loading with Polymyxin B |
| ROS | Reactive oxygen species |
| SDT | Sonodynamic therapy |
| TMOS | TCPP-doped mesoporous organo-silica (TMOS) |
| TMOS@ZIF-8 | Zeolite imidazole framework-8 sealed TCPP-doped mesoporous organo-silica |
| TCPP | Meso-tetra(4-car-boxyphenyl) porphine |
| US | Ultrasound |
| ZIF-8 | Zeolite imidazole framework-8 |

**Equipment.**

| Equipment | Manufacturer | Model |
|---|---|---|
| Transmission Electron Microscope | Japan Electron Optics Laboratory (JEOL) | JEOLJEM-1400Flash |
| Scanning Electron Microscope | JEOL | JEOL JSM-7900F |
| Fourier Transform Infrared Spectrophotometer | Beckman | Beckman Coulter DU 730 |
| High-speed Centrifuge | Thermo Fisher Scientific | Sorvall ST 16R |
| Thermostatic Magnetic Stirrer | IKA China | IKA C-MAG HS7 |
| Microplate Reader | BioTek | Synergy NEO |
| Ultrasonic Cell Disruptor | NingBo Scientz | JY92-2D |
| Mini Liposome Extruder | Avanti | 610000 |
| Zeta Potential Analyzer | Malvern | |
| Laser Scanning Confocal Microscope | Leica | LSM700 |
| UV-Visible Spectrophotometer | Shimadzu | UV-2450 |
| Bacterial Turbidity Analyzer | Beijing Meihua | MHY-28473 |
| Flow Cytometer | Agilent Technologies | FAC Symphony A5 SORP |
| Ultrasound Therapy Device | Shenzhen Dongdixin | UT1021 |
| High-throughput Tissue Grinder | NingBo Scientz | Scientz-196 |
| Mouse Tail Vein Injector | Shanghai Yuyan | |
| In Vivo Imaging System | PerkinElmer | IVIS SPECTRUM |
| Ultrasonic Cleaner | Nanjing Xianou | X025-12DT |
| X-ray Diffractometer | Bruker | D8 Advance |
| Photoelectron Spectroscopy | Thermo Fisher Scientific | Escalab 250Xi |
| Accelerated Surface Area and Porosimetry System | Micromeritics | ASAP2020 |
| Thermal Gravimetric Analyzer | Netzsch Group | STA 449C |

## Supplementary figures

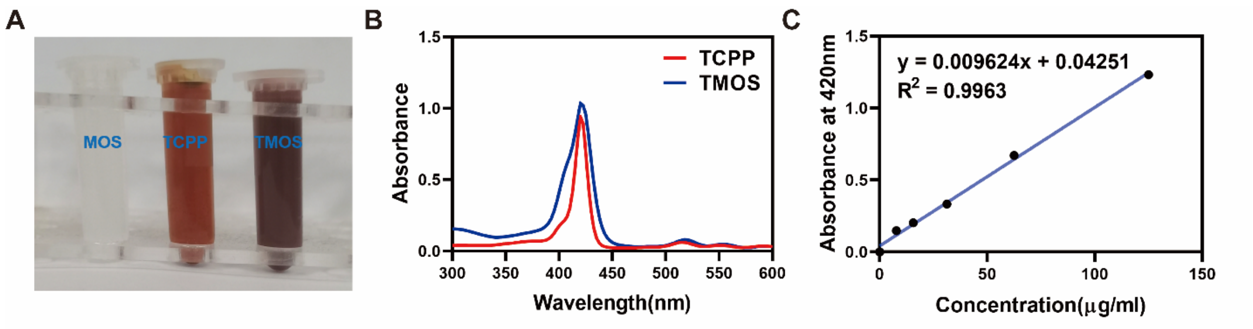


**Figure S1.** (A) Photograph of TCPP, MOS and TMOS in water. (B) UV-vis absorption of TCPP and TMOS. (C) Standard curve of TCPP with different concentrations.

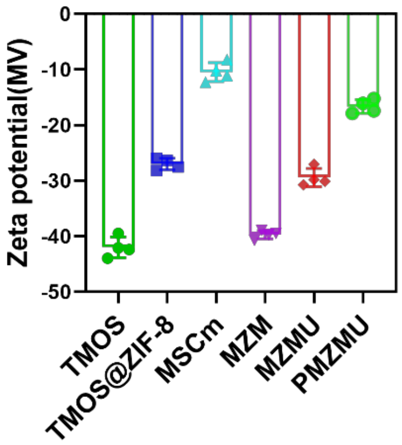


**Figure S2.** Zeta potential (mV) measurement of TMOS, TMOS@ZIF-8, MSCm, MZM, MZMU and PMZMU. Results are represented as mean ±standard deviation (n=4).

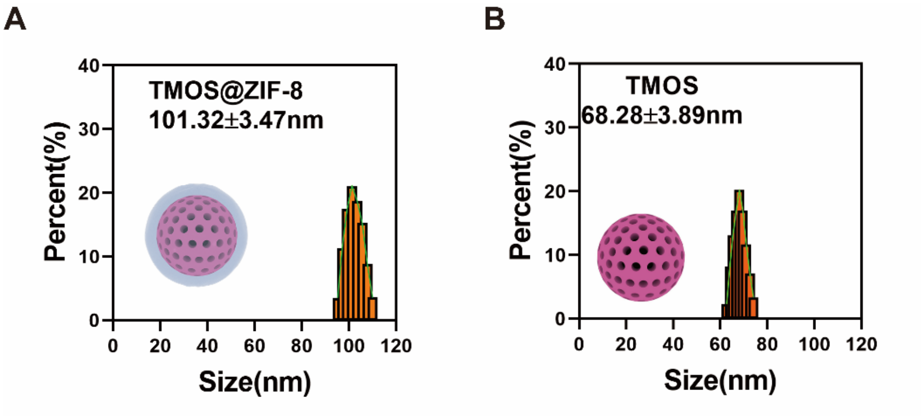


**Figure S3.** The size distribution of TMOS@ZIF-8(A) and TMOS(B) nanoparticles measured by Dynamic light scattering.

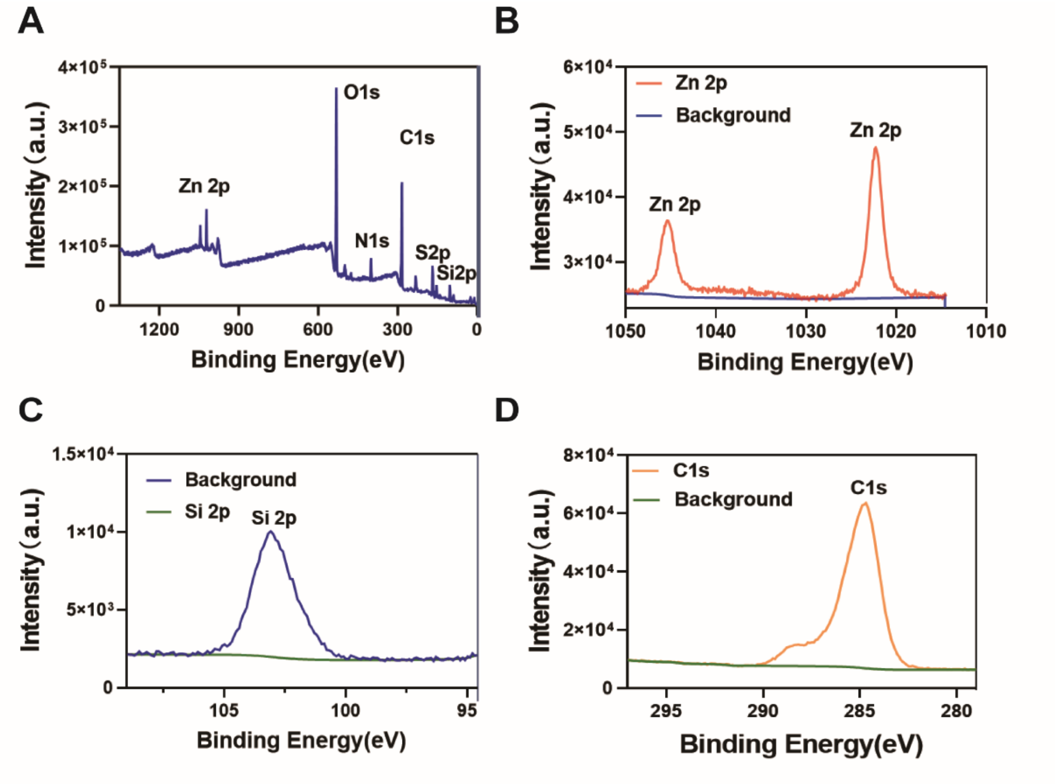


**Figure S4.** (A) XPS spectrum of TMOS@ZIF-8. High-resolution XPS spectra of Zn 2p (B), Si 2p (C) and C 1s (D) in TMOS@ZIF-8.

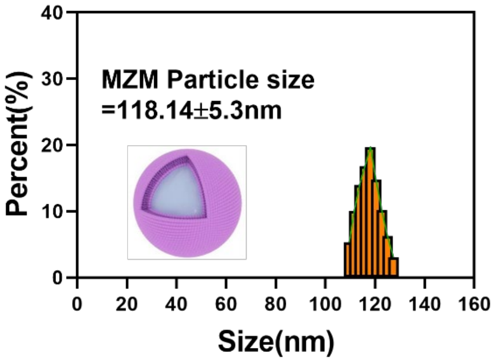


**Figure S5.** The size distribution of MZM nanoparticles measured by Dynamic light scattering.

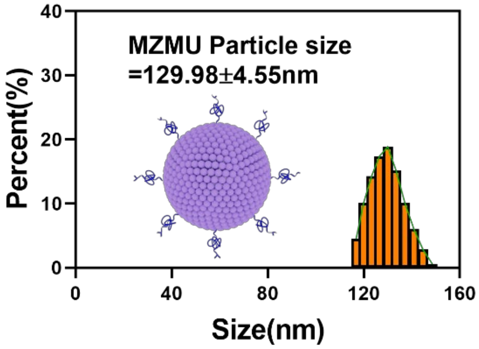


**Figure S6.** The size distribution of MZMU nanoparticles measured by Dynamic light scattering.

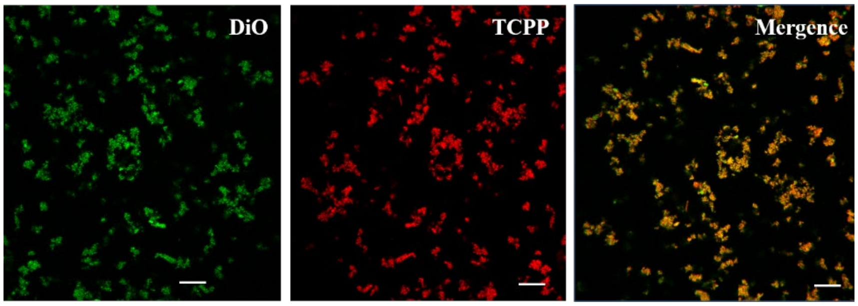


**Figure S7.** Confocal fluorescent microscopy images of MZMU (red=TMOS@ZIF-8, green=MSC membrane). Scale bar:5 μm.

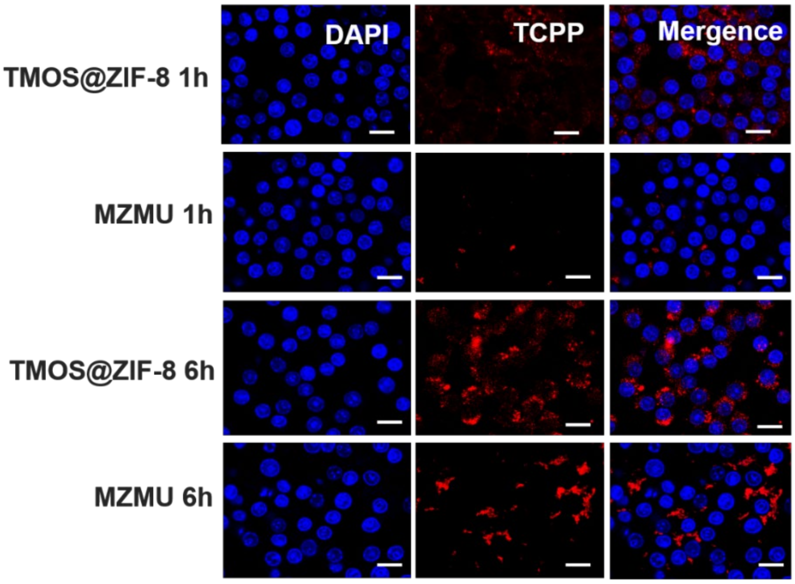


**Figure S8.** Relative fluorescence intensity of RAW264.7 cells after incubated with TMOS@ZIF-8 or MZMU for 1h and 6 h. Scale bar:20 μm.

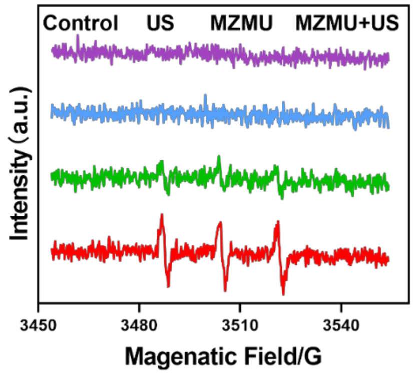


**Figure S9.** ESR spectra demonstrating [1]O2 generation of MZMU under US irradiation (1.0 MHz, 1.5 W cm$^{-2}$, 1 min).

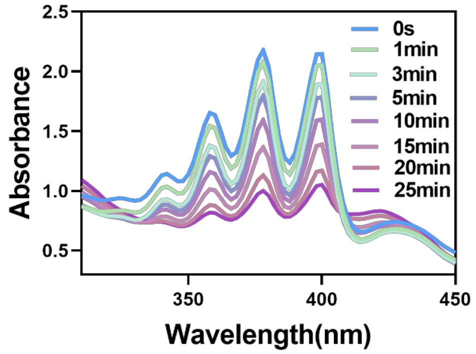


**Figure S10.** Time-dependent sonodegradation of ABDA indicating [1]O2 generated by MZMU under US irradiation (1.0 MHz, 1.5 W cm$^{-2}$).

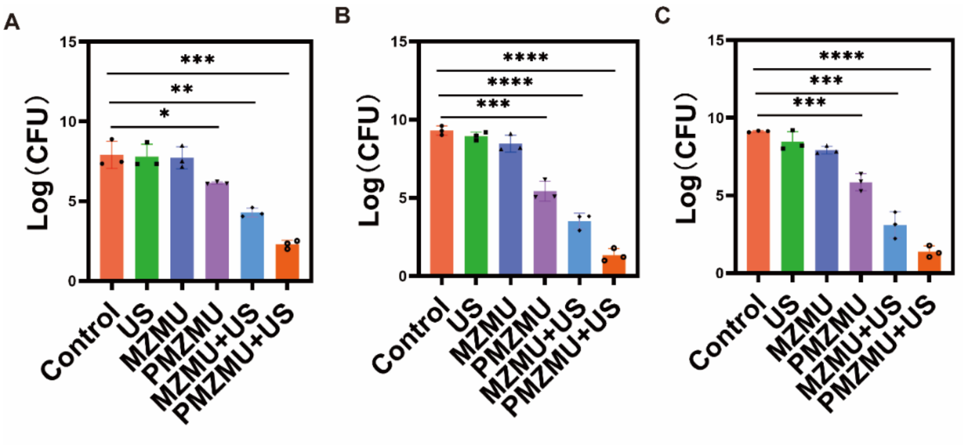


**Figure S11.** Strain counts of XDR-Ab (A), XDR-Kp (B) and XDR-E. coli (C) calculated from spread-plate assays after treated with PBS (Control), US, MZMU, PMZMU, MZMU+US and PMZMU+US. US irradiation: 1.0 MHz, 1.5 W cm$^{-2}$, 50% duty cycle, 8 min. (n = 3, *$p < 0.05$, **$p < 0.01$, ***$p < 0.001$, ****$p < 0.0001$, Data are expressed in mean ± SD).

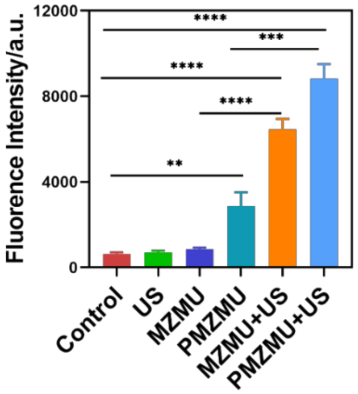


**Figure S12.**Quantitative analysis of ROS level in bacteria using flow cytometry with DCFH-DA staining. (n = 3, $**p < 0.01$, $***p < 0.001$, $****p < 0.0001$. Data are expressed in mean ± SD).

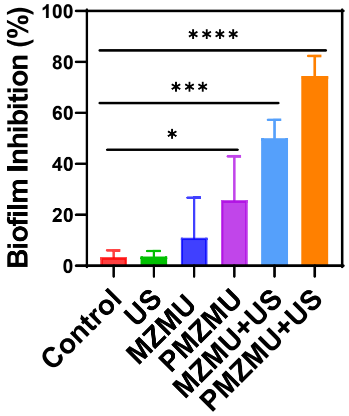


**Figure S13.** Inhibition rates of biofilms after treated with PBS (Control), US, MZMU, PMZMU, MZMU+US and PMZMU+US. (n = 3, $*p < 0.05$, $***p < 0.001$, $****p < 0.0001$. Data are expressed in mean ± SD).

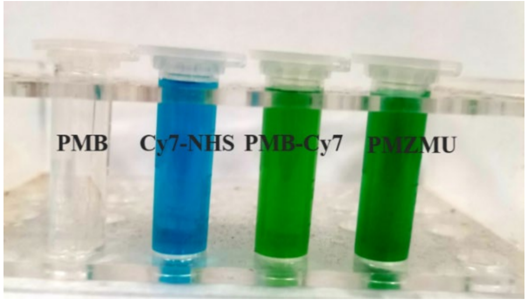


**Figure S14.** Photographs of PMB, Cy7-NHS, PMB-Cy7 and PMZMU (loading with PMB-Cy7) in water.

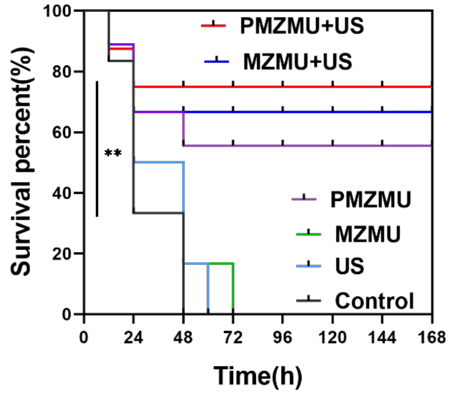


**Figure S15.** Percentage survival of pneumonia mice treated with saline (Control), US, MZMU, PMZMU, MZMU+US, and PMZMU+US (2 mg $kg^{-1}$ PMB). (n = 6, ***p* < 0.01).

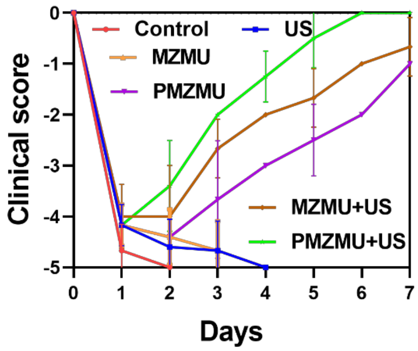


**Figure S16.**Clinical symptom scores of mice after treated with saline (Control), US, MZMU, PMZMU, MZMU+US, and PMZMU+US (n = 6).

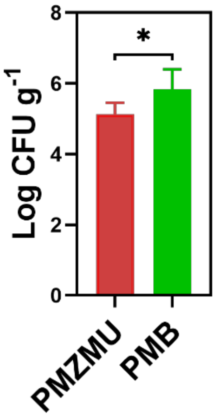


**Figure S17.** Bacterial loads in lungs of mice after treated with PMZMU and free PMB. (n = 6, **p* < 0.05. Data are expressed in mean ± SD).

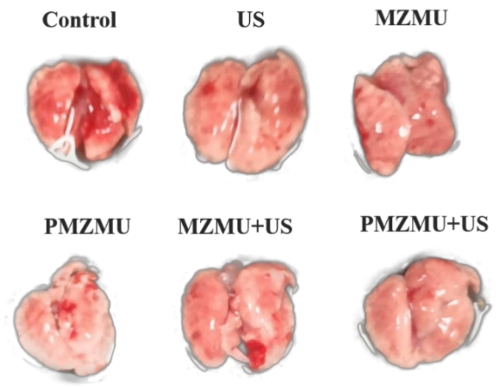


**Figure S18.** Photograph depict gross pathology of representative lungs harvested after various treatments.

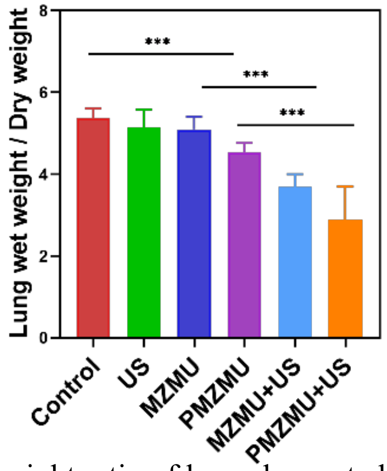


**Figure S19.**Wet weight/dry weight ratio of lungs harvested after various treatments. (n = 6, ***$p < 0.001$. Data are expressed in mean ± SD).

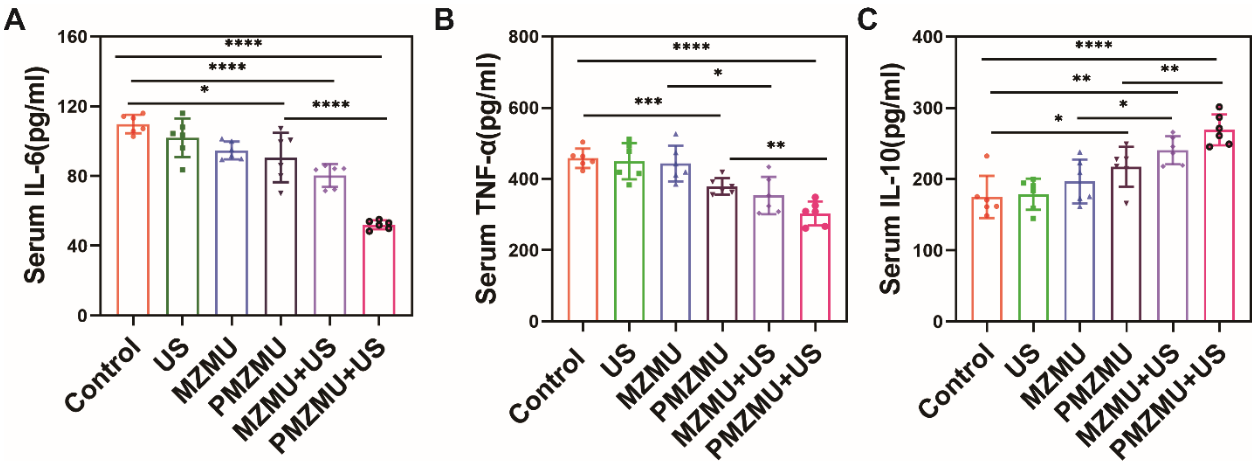


**Figure S20.**Serum levels of IL-6(A), TNF-α (B) and IL-10 (C) collected after various treatments. (n = 6, *$p < 0.05$, **$p < 0.01$, ***$p < 0.001$, ****$p < 0.0001$. Data are expressed in mean ± SD).

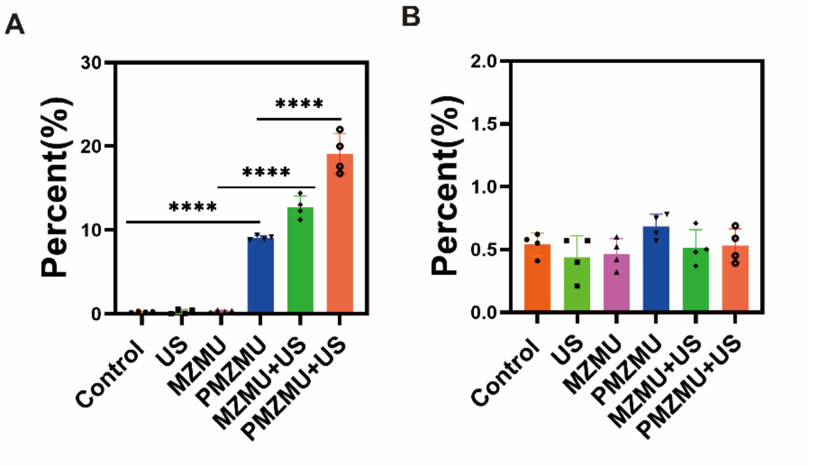


**Figure S21.**Quantitative analyses of CD206 (A) and CD86 (B) expression in macrophages (RAW 264.7) cells after different treatments. CD206 present $M_2$ polarization, CD86 present $M_1$ polarization. (n = 4, ****$p < 0.0001$. Data are expressed in mean ± SD).

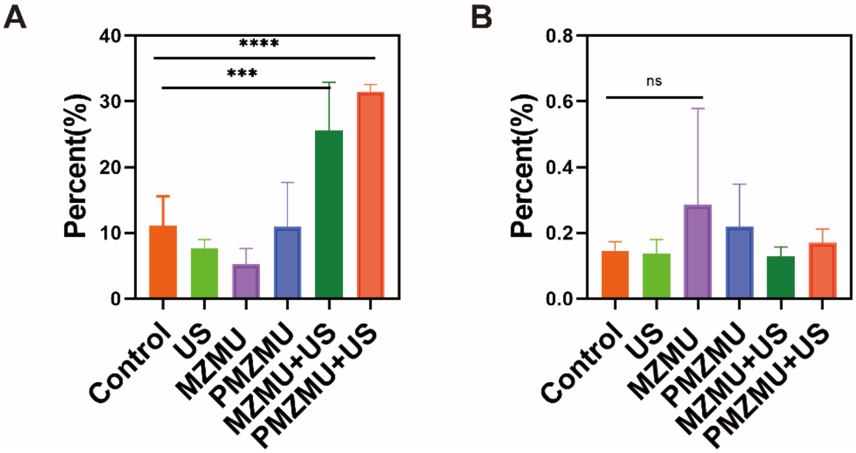


**Figure S22.**Quantitative analyses of the percentage of (A) M2-type macrophages ($CD45^+CD11b^+F4/80^+CD206^+$) and (B) $M_1$-type macrophages ($CD45^+CD11b^+F4/80^+CD86^+$) in the spleens of mice from different treatment groups. (n=6, ***$p < 0.001$, ****$p < 0.0001$, n. s means no significance. Data are expressed in mean ± SD).

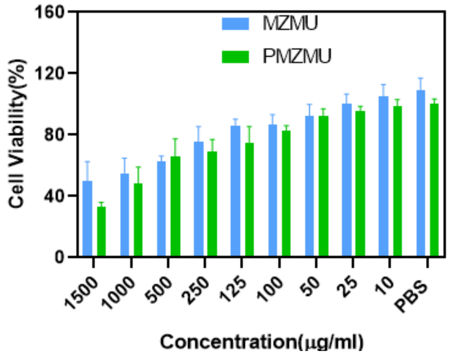


**Figure S23.** Cytotoxicity of human bronchial epithelial cells (BEAS-2B) after being treated by different concentrations of MZMU and PMZMU for 24 h. Results are represented as mean ± standard deviation (n=6).

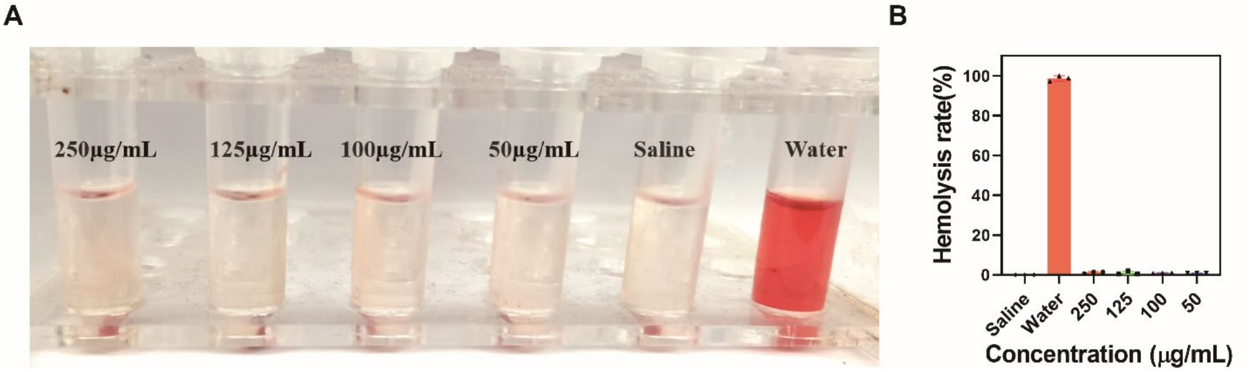


**Figure S24.** (A) Representative photograph and (B) relative hemolysis rate of RBC treated with different concentrations of PMZMU (50, 100, 125, 250 μg $mL^{-1}$), using water as a positive control and saline as a negative control. Results are represented as mean ± standard deviation (n = 3).

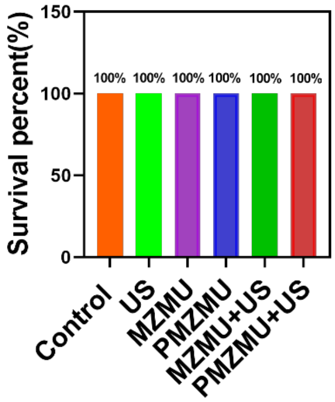


**Figure S25.** Survival rate of mice treated with saline, US, MZMU, PMZMU, MZMU +US, PMZMU+US during 28 days. (n = 3).

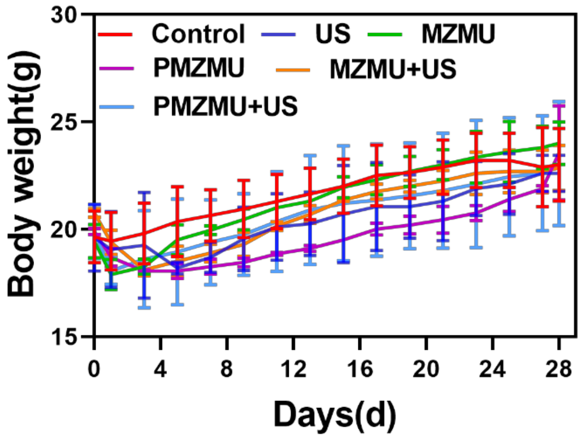


**Figure S26.** The body weight change of mice treated with saline (Control), US, MZMU, PMZMU, MZMU +US, PMZMU+US during 28 days. (n = 3)

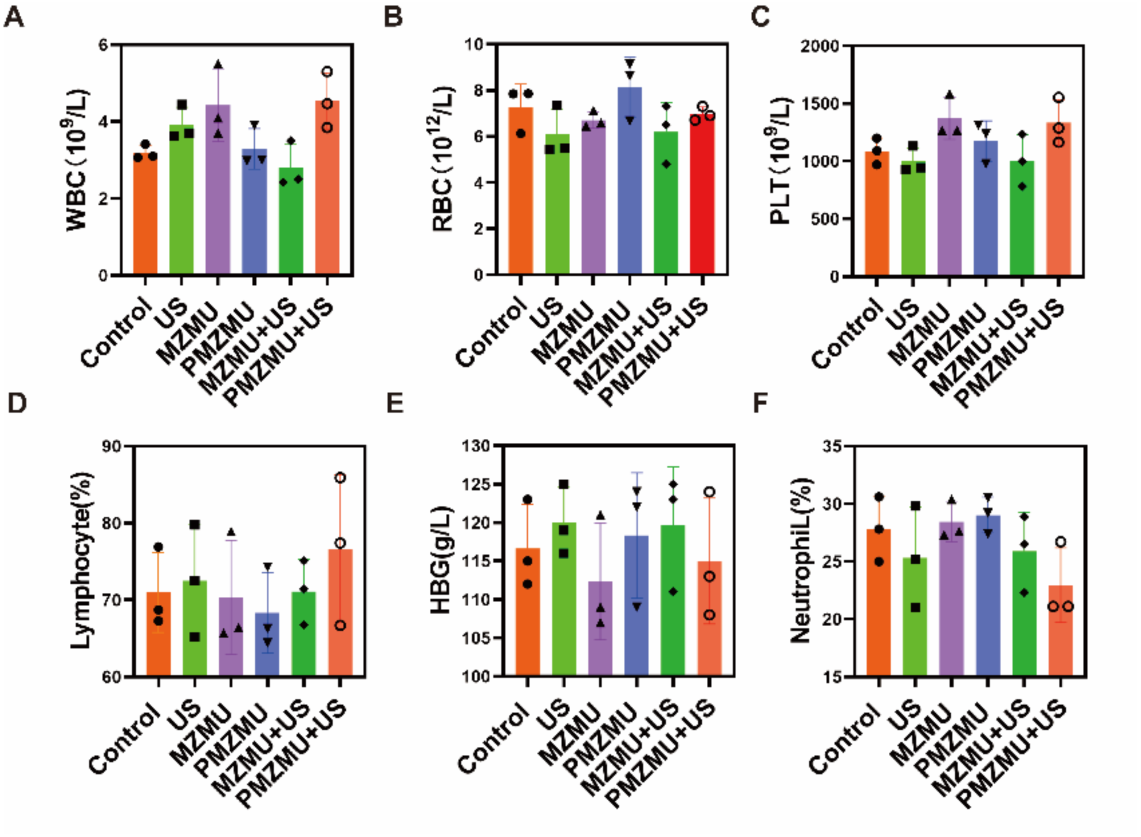


**Figure S27.** The hematology analysis results of mice in different treatment groups on day 28 after administration. (A) White blood cells (WBC), (B) Red blood cells (RBC), (C) Platelets (PLT), (D) Lymphocytes, (E) Hemoglobin (HBG), (F) Neutrophil proportion. (n = 3).

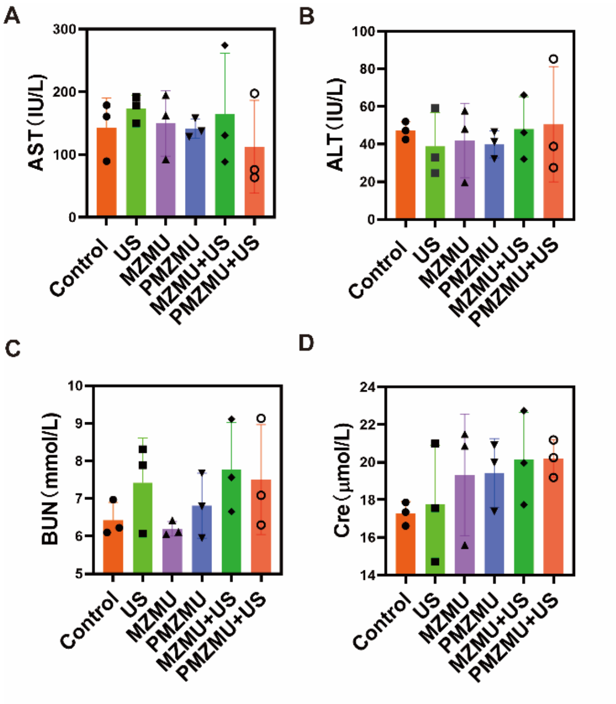


**Figure S28.** Blood biochemical analysis results of mice in different treatment groups on day 28 after administration. (A) Aspartate aminotransferase (AST), (B) Alanine aminotransferase (ALT), (C) Blood urea nitrogen (BUN), (D) Creatinine (Cre). (n = 3).

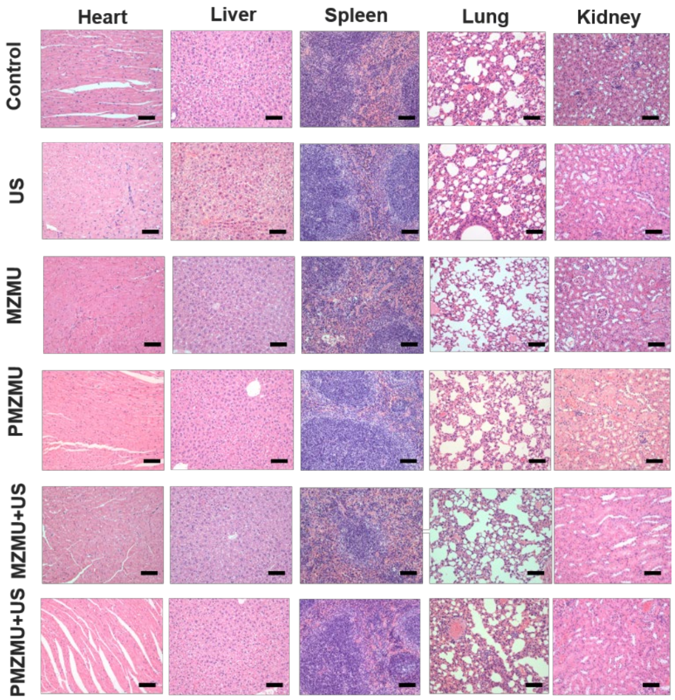


**Figure S29.** H&E staining of major organs sections of mice at day 28 after different treatments. Scale bar:50μm.

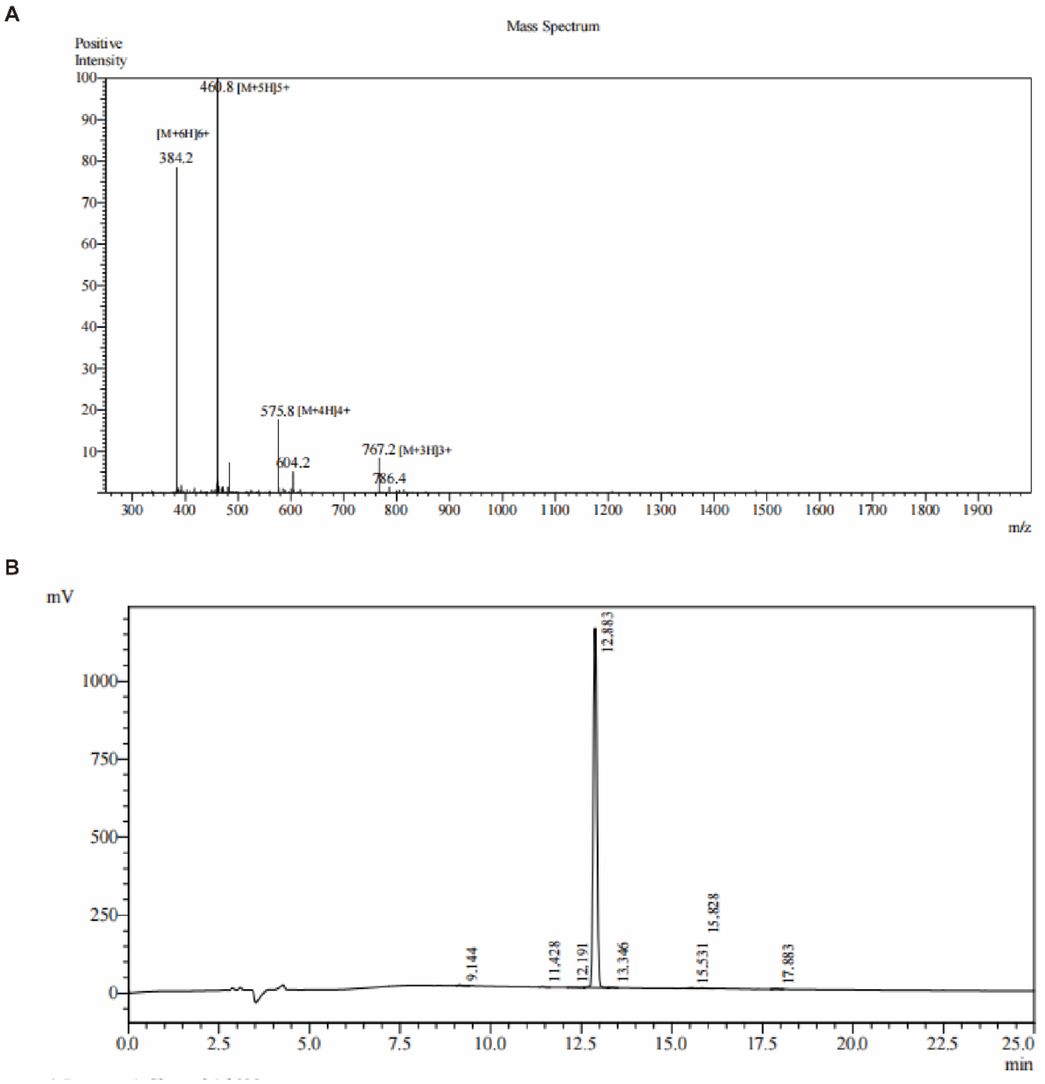


**Figure S30.** High performance liquid chromatography and mass spectrometry of $UBI_{29-41}$. (A) Mass spectrometry shows $UBI_{29-41}$ with the molecular weight of 2298.64 Da. (B) High performance liquid chromatography of $UBI_{29-41}$.

**Table S1.** Evaluation of the Minimum Inhibitory Concentration (MIC) of MZMU, PMZMU nanoparticles and free PMB.

| **Bacterial strains** | **MIC (μg mL$^{-1}$)** | | |
|---|---|---|---|
| | MZMU | PMZMU | PMB |
| XDR-AB | N | 16 | 32 |
| XDR-PA | N | 4 | 8 |
| XDR-E. coli | N | 16 | 16 |
| XDR-Kp | N | 4 | 16 |

N: No Inhibition

**Table S2.** Drug sensitivity test of four clinical strains

| **Antibiotics** | **MIC (μg mL$^{-1}$)** | | | |
|---|---|---|---|---|
| | XDR-AB | XDR-PA | XDR-Kp | XDR-E. coli |
| Ampicillin | R (>=32) | R (>=32) | R (>=32) | R (>=32) |
| Cefazolin | R (>=64) | R (>=64) | R (>=64) | R (>=64) |
| Cefotetan | R (>=64) | R (>=64) | R (>=64) | R (>=64) |
| Levofloxacin | R (>=8) | R (>=8) | R (>=8) | R (11) |
| Sulbactam/Ampicillin | R (>=32) | R (>=32) | R (>=128) | R (>=32) |
| Cotrimoxazole | R (>=320) | R (>=320) | R (>=320) | R (>=320) |
| Amikacin | R (>=64) | R (>=64) | R (>=64) | R (>=64) |
| Cefepime | R (>=64) | R (>=64) | R (>=64) | R (>=64) |
| Imipenem | R (>=16) | R (>=16) | R (>=16) | R (>=16) |
| Ceftazidime | R (>=64) | R (>=64) | R (>=64) | R (>=64) |
| Piperacillin/Tazobactam | R (>=128) | R (>=128) | R (>=128) | R (>=128) |
| Gentamycin | R (>=16) | R (>=16) | R (>=16) | R (>=16) |
| Tobramycin | R (>=16) | R (>=16) | R (>=16) | R (>=16) |
| Ciprofloxacin | R (>=4) | R (>=4) | R (>=4) | R (>=4) |
| Cefotadine/Avibactam | R (>=64) | 20(KB) | 24(KB) | R (>=64) |
| Polymyxin B | / | S (0.5) | / | S (0.5) |
| Tigecycline | S (0.38) | | | |

**Table S3.** Detailed information of the source patients.

| Name | Gender | Age | Diagnosis | Treatment | Bacteria strain | Source sample | Prognosis |
|---|---|---|---|---|---|---|---|
| Ge xx | Male | 75 | urinary tract infection | Cefoperazone sulbactam | XDR-E. coli | urine | better |
| Sun xx | Male | 92 | bacterial pneumonia | Ceftazidime Avibactam | XDR-Kp | sputum | death |
| Ding xx | Male | 75 | bacterial pneumonia | Levofloxacin | XDR-PA | sputum | better |
| Zhou xx | Male | 80 | bacterial pneumonia | Tigecycline and Meropenem | XDR-Ab | sputum | better |
